# Using Matrix and Tensor Factorizations for the Single-Trial Analysis of Population Spike Trains

**DOI:** 10.1371/journal.pcbi.1005189

**Published:** 2016-11-04

**Authors:** Arno Onken, Jian K. Liu, P. P. Chamanthi R. Karunasekara, Ioannis Delis, Tim Gollisch, Stefano Panzeri

**Affiliations:** 1 Neural Computation Laboratory, Center for Neuroscience and Cognitive Systems @UniTn, Istituto Italiano di Tecnologia, Rovereto, Italy; 2 Department of Ophthalmology, University Medical Center Goettingen, Goettingen, Germany; 3 Bernstein Center for Computational Neuroscience Goettingen, Goettingen, Germany; 4 Center for Mind/Brain Sciences, University of Trento, Rovereto, Italy; 5 Department of Biomedical Engineering, Columbia University, New York, New York, United States of America; University of Tübingen and Max Planck Institute for Biologial Cybernetics, GERMANY

## Abstract

Advances in neuronal recording techniques are leading to ever larger numbers of simultaneously monitored neurons. This poses the important analytical challenge of how to capture compactly all sensory information that neural population codes carry in their spatial dimension (differences in stimulus tuning across neurons at different locations), in their temporal dimension (temporal neural response variations), or in their combination (temporally coordinated neural population firing). Here we investigate the utility of tensor factorizations of population spike trains along space and time. These factorizations decompose a dataset of single-trial population spike trains into spatial firing patterns (combinations of neurons firing together), temporal firing patterns (temporal activation of these groups of neurons) and trial-dependent activation coefficients (strength of recruitment of such neural patterns on each trial). We validated various factorization methods on simulated data and on populations of ganglion cells simultaneously recorded in the salamander retina. We found that single-trial tensor space-by-time decompositions provided low-dimensional data-robust representations of spike trains that capture efficiently both their spatial and temporal information about sensory stimuli. Tensor decompositions with orthogonality constraints were the most efficient in extracting sensory information, whereas non-negative tensor decompositions worked well even on non-independent and overlapping spike patterns, and retrieved informative firing patterns expressed by the same population in response to novel stimuli. Our method showed that populations of retinal ganglion cells carried information in their spike timing on the ten-milliseconds-scale about spatial details of natural images. This information could not be recovered from the spike counts of these cells. First-spike latencies carried the majority of information provided by the whole spike train about fine-scale image features, and supplied almost as much information about coarse natural image features as firing rates. Together, these results highlight the importance of spike timing, and particularly of first-spike latencies, in retinal coding.

## Author Summary

The ever growing size of neural populations simultaneously recorded in electrophysiological experiments calls for urgent analytical progress in understanding how to compactly describe all sensory information present both in the spatial and temporal structure of single-trial neural population activity. Here we show the power of analytical methods, termed space-by-time tensor factorizations, which detect groups of simultaneously coactive neurons and the temporal profiles of their coactivation. By validation on simulated data and on retinal recordings, we show that the tensor decomposition performs competitively compared to other techniques both in terms of data robustness and ability to find informative patterns across diverse stimuli. We show that this method can determine the spatial and temporal resolution of neural population codes, and find which spatial or temporal components of neural responses carry information not available in other aspects of the population code. When applied to experimental data, the method demonstrates the importance of first-spike latencies in retinal population coding of visual images, particularly for decoding fine spatial details of natural images from population activity. This work shows that this methodology can improve our knowledge of population coding by allowing the discovery of informative spatial and temporal firing patterns in populations of simultaneously recorded neurons.

## Introduction

In response to sensory stimuli, neural circuits produce coordinated patterns of neural population activity [1]. Understanding how information about sensory features is encoded in the firing patterns of neural populations and how other neural circuits may decode this information is crucial for understanding functions such as sensation and perception.

Two dimensions of neural representations are important for characterizing a neural code. The first is defined by space: the diversity of stimulus tuning of individual neurons at different spatial locations, and the synchrony in their activity, shape how populations encode information [2–6]. The second dimension is defined by time: neuronal responses evolve over time, and the temporal structure of neural activity can only be neglected at the cost of losing considerable information [7–17]. Simultaneous recordings of large populations are beginning to show how the spatial and temporal dimensions interact to form neural representations. An emerging result from these studies has been that, perhaps because of constraints imposed by the hard-wiring of the neural circuitry, neural populations express a limited range of stereotyped spike timing patterns [18–22] made of groups of neurons that tend to fire close together in time, with the relative strength and timing of different patterns encoding information about the stimulus features [22, 23].

A widely used non-parametric approach for characterizing neural population spike timing patterns is to measure the probability of observing every possible “spike word” reporting the presence or absence of spikes in a sequence of short windows [24–27] and then comparing these distributions across experimental conditions using information theoretic measures [28]. This method can capture all information present in spike trains, including the effect of correlations at all orders. However, because the number of possible spike words grows exponentially with the number of neurons, spike word analyses are either applied to very small populations of few neurons when their time structure is taken into account, or is limited to a few tens of neurons when the temporal structure of responses is neglected and only simultaneous firing across neurons is considered [29]. The lack of scalability of this approach precludes its utilization for analysis of the large-scale recordings that are emerging now and will become even more important in the near future.

How to extract a biologically meaningful and scalable representation of neural population spike trains in space and time remains an open problem. Such a representation should satisfy many requirements. First, because nervous systems make decisions in single trials, it should capture information in single trial spike trains. Second, it should capture most or all information about stimuli with a small number of parameters. Third, the basis functions used to describe single-trial neural activity should be interpretable biologically: in particular, it should decompose neural activity into the constituent stereotyped patterns of firing observed in the data. Fourth, if these recurring patterns express to some extent hard-wired aspects of circuitry, we would expect that stereotyped patterns extracted by this representation from responses to some stimuli are then found also in responses to other stimuli.

Current methods for finding low-dimensional representations of neural activity [30–34] such as Principal Component Analysis (PCA), Independent Component Analysis (ICA), or Factor Analysis (FA) are usually applied to firing rate only (neglecting the temporal structure of spike trains) or to trial-averaged data (to avoid the confounding effects of trial-to-trial spiking), but do not usually explicitly identify the temporal structure [35, 36], although in the case of Gaussian Process Factor Analysis (GPFA) some of the temporal dynamics of spike trains has been modeled as temporal correlations between latent variables. Here we investigate the potentials for describing single-trial spatiotemporal firing patterns of two potentially useful methodological approaches (and of their intersection). The first direction is to investigate whether so-called tensor approaches that decompose the spatiotemporal spike patterns under the assumption that they can be factored in space and time may provide greater robustness and biological interpretability of results. Some of these approaches have proved useful in neuroimaging analysis [37–48], yet their effectiveness in meeting our requests for single-trial spike trains remain untested. The second direction is to explore linear decompositions of single trial population spike trains into a sum over non-negative basis functions using non-negative coefficients, as it happens for example in Non-negative Matrix Factorization (NMF, see [49]). The non-negativity constraint potentially yields several advantages: its basis functions and coefficients are, in principle, directly interpretable as firing patterns and as their strength of recruitment in single trials; it generates sparse representations; and it can cope with non-orthogonal firing patterns such as partly overlapping ones that may be generated by neural circuits with hard-wired connectivity. Yet, the effectiveness of this constraint, and other possible ones, on single-trial spike train analysis remains largely unexplored.

In this article we explore the potentials of tensor factorizations in space and time, and of non-negativity constraints, for spike train analysis by applying these techniques to both simulated spike trains and simultaneous electrophysiological recordings of populations of retinal ganglion cells (RGCs). Comparing with other possible data reduction strategies, we found that all considered tensor decompositions yielded superior stimulus decoding of population spike times. Within tensor decompositions, the constraints of orthogonality among the extracted firing patterns led to better decoding performance. Tensor decompositions with a non-negativity constraint (which decompose the spatiotemporal patterns into groups of neurons that fire simultaneously and provides temporal basis functions for their activation) led to very good (though not optimal) decoding performance and to excellent interpretability, sparseness and robustness of detection of firing patterns. We also studied how to use this representation to determine the specific contribution of precise spike timing to population codes made of tens of cells. By application of tensor methodology to retinal ganglion cells, we were able to describe how spike timing contributes both to the population coding of flashed images and of dynamic natural movies.

## Results

### Single-trial population spike train decompositions for detecting patterns in neural activity

**Fig 1:**
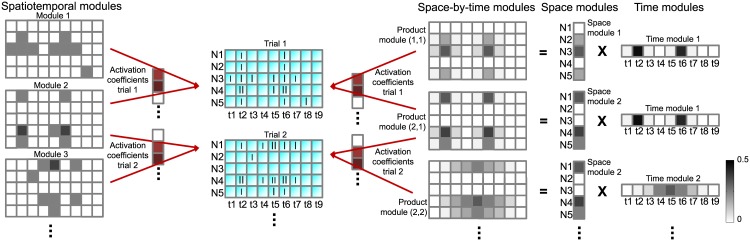
Illustration of matrix-based spatiotemporal and tensor-based space-by-time decompositions of single-trial population spike trains. In this cartoon, two single-trial spike trains of a population of neurons (N1 to N5) binned into time bins (t1 to t9) are shown in cyan. The spatiotemporal decomposition (shown on the left) decomposes the population spike trains into spatiotemporal modules (three in this example) using a set of three activation coefficients (shown in red) in each trial. The space-by-time decomposition of the same data (shown on the right) factorizes the single-trial population spike trains into a set of temporal modules, a set of spatial modules and a set of single-trial activation coefficients. The temporal modules describe the temporal activity patterns that are present in the data. The spatial modules describe which groups of neurons fire together and their relative activation levels. The activation coefficients describe which temporal patterns and which groups of neurons are combined in order to form each single-trial population response.

We investigated how to learn low-dimensional yet sensory-information-rich representations of single-trial neural population spike patterns in terms of recurring spatiotemporal firing patterns. Our starting point is a matrix-based spatiotemporal representation of neural activity as a spike count word matrix (Fig 1), where hereafter ‘temporal’ refers to time of activity relative to stimulus-onset and ‘spatial’ refers to neuron identity, i.e. which neurons are active. We then use a linear decomposition of a spike count word matrix into a sum over a set of basis functions (referred to as modules or components).

We evaluated two possible decomposition variants. The first variant (Fig 1, left), which we call matrix-based or spatiotemporal decomposition and we define in Eqs (1) and (2), identifies a set of spatiotemporal modules as matrices that specify the time bins at which each individual neuron fires. The identified modules are linearly combined on each trial using scalar activation coefficients to reconstruct the original spike count word matrix. This decomposition can be formulated as a factorization of the spike count word matrix into two matrices: one containing the extracted spatiotemporal modules and another one containing the coefficients activating them in single trials.

The second variant (see Fig 1, right panel, and Eqs (4)–(6)), called tensor-based or space-by-time decomposition, additionally factorizes the space and time dimensions by identifying separate sets of modules for space (i.e. distribution of firing across neurons) and time (i.e. temporal profiles of firing). The space-by-time modules are then expressed as products of the spatial and temporal modules. This decomposition can be formulated in a general way as a tensor factorization known as Tucker-2 decomposition [50, 51]. The approach factorizes the tensor composed of all spatiotemporal trials into two factor matrices corresponding to spatial and temporal modules and a core tensor corresponding to the activation coefficients that linearly combine the spatial and temporal modules in single trials to approximate the spike count word matrix.

The potential advantage of the spatiotemporal decomposition is its generality, as it can approximate complex firing rate patterns of arbitrary shape (without the assumption of a separate structure in space and time). Its potential drawback is the high-dimensionality of the basis functions which may make it difficult to learn spatiotemporal patterns from noisy and limited neural data. The potential disadvantage of the space-by-time decomposition is that it may not be able to capture all types of patterns with a small number of parameters. However, the simplicity and low-dimensionality of its basis functions make the space-by-time method potentially more robust.

A linear decomposition of the above types is generally not unique. To improve the uniqueness properties of the decompositions and also to facilitate interpretability of the extracted factors, it is necessary to impose constraints on the factors’ structure or statistical relationship. Here we explored the effect of such constraints.

In the matrix spatiotemporal factorization, the first type of constraint is non-negativity (i.e. non-negative basis functions and non-negative coefficients) and the corresponding matrix decomposition is known as Non-negative Matrix Factorization (NMF [49]). NMF has been widely used in machine learning, for image analysis, and for a variety of biological applications–including prominently the analysis of muscle synergies from electromyographic recordings [52], but it has been seldom explored for spike train analysis. Imposing the constraint of non-negativity is well known to decompose the input dataset into its “parts”, thereby producing sparse low-dimensional representations [49]. Given that spike trains are non-negative, the constraint of non-negativity seems particularly natural for the description of non-negative single-trial spike trains, as it potentially enables us to interpret the basis function of this decomposition as recurring population spike patterns and the coefficients of this decomposition as the strength by which the stereotypical patterns are recruited on a single trial. Another type of constraint involves imposing a statistical relationship on the extracted factors. The most common such constraints are orthogonality and statistical independence between the factors and the corresponding matrix factorization methods are Principal Component Analysis (PCA [32, 33, 53–55]) and Independent Component Analysis (ICA [32, 33]) respectively. Therefore, for spatiotemporal matrix decompositions, we applied spatiotemporal NMF, PCA, ICA and the closely related Factor Analysis (FA [56–58]) which, in contrast to the other methods, assumes a latent variable model.

For the tensor space-by-time representations, we enforced such constraints to the Tucker-2 decomposition. Application of non-negativity constraints yields a non-negative tensor decomposition here referred to as space-by-time NMF and also known as non-negative Tucker-2 [59, 60]. Orthogonality constraints yield a tensor decomposition known as orthogonal Tucker-2, which can be understood as a tensor generalization of PCA [50, 51]. We also compared the Tucker-2 decomposition methods with another tensor factorization, known as Parallel Factor Analysis (PARAFAC, [61]) or Canonical Decomposition (CANDECOMP, [62]), allowing no interactions between the factors. This constraint implies that the core tensor is the identity tensor [50] and all factors have the same number of modules. From the PARAFAC family, we applied the Bayes Poisson Factor method which has been shown to be particularly effective in decomposing count data [63]. For a comparison with generalizations of ICA to tensor factorizations, see [64].

### Performance of different single-trial spike train decomposition methods in correctly retrieving the shape of firing patterns in simulated datasets

**Fig 2:**
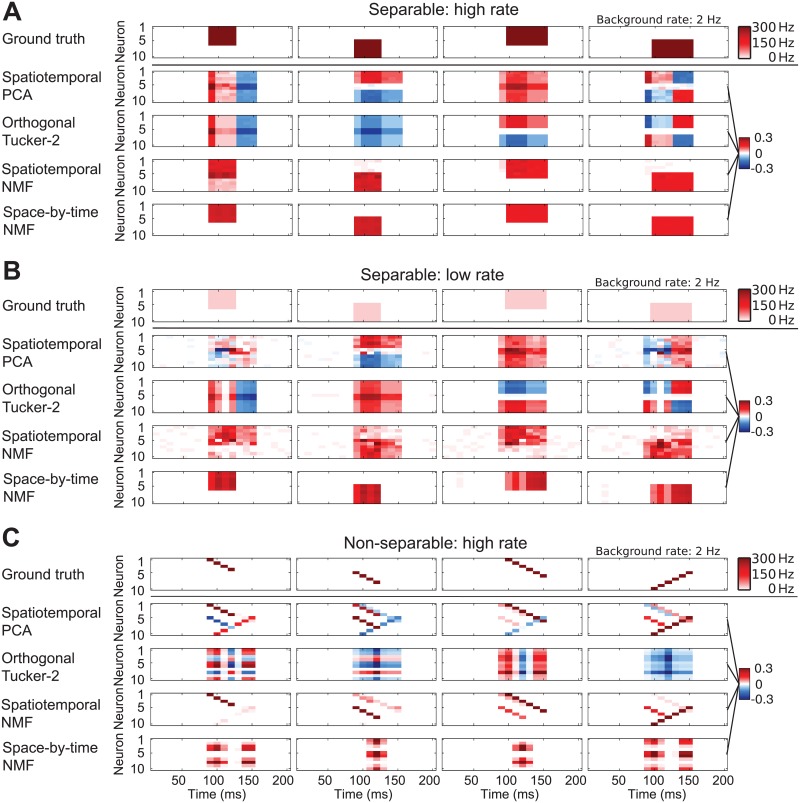
Recovering firing patterns from simulated data. To illustrate how different decompositions work, we show how a dataset made of 900 trials of four randomly mixed “ground truth” firing patterns may be decomposed by different methods. (A) A case when the ground truth modules can be factorized into space and time. Top row: Four ground truth modules for generating spike trains. Inhomogeneous Poisson spike trains are generated with a background rate (white) and a stronger foreground rate (red). The red blocks fire with high SNR (300 Hz vs. a background rate of 2 Hz). Each row shows the modules that were recovered by the denoted method. Only spatiotemporal NMF and space-by-time NMF manage to recover the true underlying blocks. (B) As in panel A but with ground truth patterns made of blocks with lower SNR (30 Hz vs. background rate of 2 Hz). In this case, spatiotemporal NMF cannot recover the underlying blocks as well anymore. (C) A case of decomposition of high firing rate patterns that are not separable in space and time. Spatiotemporal NMF best identifies the ground truth patterns.

To illustrate and evaluate the capabilities of each low-dimensional representation, we first tested them on synthetic population spike trains. We generated Poisson spike trains with “ground truth” spatiotemporal patterns consisting of one or more brief periods of strong firing activity (much elevated over the background level of firing rate) for a selected subgroup of neurons (Fig 2A–2C top rows). These patterns had the form of four partly overlapping blocks corresponding to different neurons having increased firing rates either at the same time or at subsequent times. We designed these patterns to be either factorable (Fig 2A and 2B) or non-factorable (Fig 2C) in space and time. Notably, owing to their overlap all of these patterns are non-orthogonal and statistically dependent, as may be expected from spike patterns generated across different conditions from hard-wired neural circuits [22, 23]. We then used various analysis methods (PCA, orthogonal Tucker-2, spatiotemporal NMF, space-by-time NMF shown in Fig 2; ICA, FA, Bayes Poisson Factor shown in S1 Fig) to decompose these data and we considered whether the basis functions retrieved were similar to the “ground truth” firing patterns. We asked the methods to decompose the data into four modules (which equaled the true number of patterns used to generate the data). Specifically, the space-by-time factorization of these data was performed with two spatial and two temporal modules.

We first considered a case in which the patterns of elevated activity to be detected were constructed with a higher signal-to-noise ratio (SNR), as the periods of elevated activity had a very high firing rate of 300 Hz (Fig 2B) against a background rate of 2 Hz. (We defined the SNR of the firing pattern to be detected as the ratio between its firing rate and the background firing rate). We applied the different decompositions to population spike patterns, simulated according to randomly mixed firing rate profiles, i.e. each of the four patterns had a 50% chance of being present in a trial. We then quantified the similarity between the ground truth and the recovered modules (shortened as “module recovery similarity” hereafter) by calculating the geodesic similarity between the ground truth patterns and the patterns found by the various methods over 30 simulations of 900 trials each (c.f. S1 Text, Section “Quantification of similarity between modules”).

We found that spatiotemporal NMF, space-by-time NMF and Bayes Poisson Factor recovered patterns that were close to the ground truth patterns (Fig 2A, S1A Fig, showing patterns recovered from a single run with 900 trials, module recovery similarity 88.25%, 98.8% and 85.9% respectively). Spatiotemporal PCA, ICA, FA and orthogonal Tucker-2, on the other hand, failed to identify the original patterns because these patterns were non-orthogonal, not statistically independent, and did not fit the probabilistic model of FA (Fig 2A, S1A Fig, module recovery similarity < 60%). It should be noted that, even though PCA and orthogonal Tucker-2 did not recover the original “ground truth” modules, they did recover components that represent orthogonalized ground truth modules. In principle, the components that these methods recover could be rotated to recover the original ground truth modules, but the required transformation of the PCA and orthogonal Tucker-2 components is generally not known.

We then generated (Fig 2B) other Poisson firing patterns that had the same spatiotemporal shape as in the previous case, but had a much lower elevated firing of 30 Hz (and thus had a 10-fold decrease in SNR) than those considered above. In these conditions of lower SNR (Fig 2B) spatiotemporal NMF did not recover the modules as well anymore (module recovery similarity 76.7%), whereas space-by-time NMF still achieved good recovery performance (module recovery similarity 86.8%). The performance of Bayes Poisson Factor was in between (S1B Fig, module recovery similarity 80.4%). The reason why space-by-time NMF operated more robustly than spatiotemporal NMF is that the former (using modules that are factorized separately in space and time) utilizes a simpler set of modules defined by fewer parameters.

We next generated (Fig 2C) firing patterns made of a sequential activation of neurons within each pattern. These patterns are non-separable in space and time. Also in this case, orthogonal Tucker-2, spatiotemporal ICA and FA still failed to produce modules that corresponded to the original ground truth firing patterns (Fig 2C, S1C Fig, module recovery similarity < 62%). Spatiotemporal PCA did somewhat better in this case (Fig 2C, module recovery similarity 75.5%). Spatiotemporal NMF managed to recover the ground truth patterns relatively well (Fig 2C, module recovery similarity 80.4%) and significantly better than spatiotemporal PCA (*p* < 0.001, two-tailed t-test), but space-by-time NMF (Fig 2C) failed to do so because the spatiotemporal factorization assumption is violated in this case (module recovery similarity < 61%). Of course the non-separable spatiotemporal patterns would still be well described by a space-by-time decomposition with a number of modules larger than the number of ground truth modules, and in such a case the spatial and temporal basis functions would be made of single neurons and single time points respectively. This shows that spatiotemporal and space-by-time methods have both strengths and weaknesses: the additional space-by-time factorization assumption increases the robustness of the methods, but also prevents detection of the correct spatiotemporal patterns if the patterns are non-separable in space and time unless a very large basis function set is used (and thus little advantage is achieved in terms of reducing dimensionality).

### Comparison on simulated data of the ability of algorithms to correctly identify stereotyped patterns across different stimulus conditions

**Fig 3:**
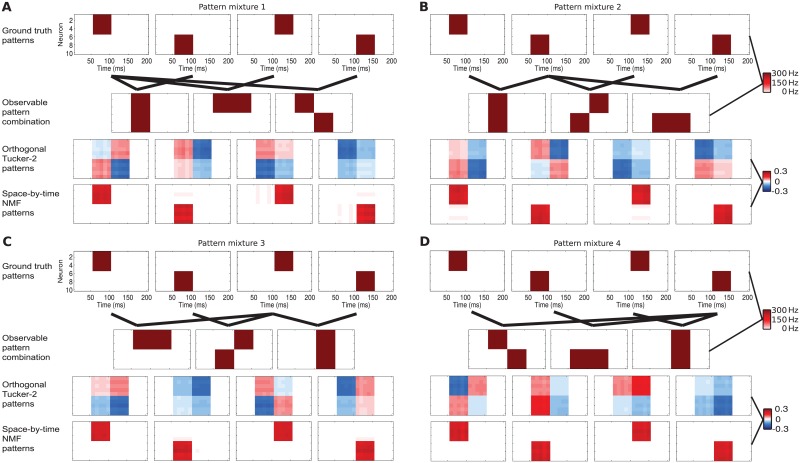
Patterns retrieved by orthogonal Tucker-2 and by space-by-time NMF from simulated data. We show how 4 different mixed patterns affect the modules retrieved by orthogonal Tucker-2 and by space-by-time NMF. Each of the panels (A to D) corresponds to one pattern mixture. (Top row) Patterns for spike train generation are plotted with the same conventions as in Fig 2A. Ground truth patterns are not overlapping. (Second row) Ground truth patterns are mixed in different ways to compose the observable patterns. (Third row) Patterns recovered by orthogonal Tucker-2. The patterns strongly depend on the specific mixture, even though the ground truth patterns are always the same. (Fourth row) Patterns recovered by space-by-time NMF. The method manages to identify the correct ground truth modules.

The above simulations suggest that modules detected with non-negative decomposition patterns are more directly interpretable in terms of the firing patterns that make up the spike train dataset. As mentioned above, due to constraints of hard-wired connectivity, it is likely that the same population, when tested with different stimuli will still emit the same type of firing patterns though with some differences in the relative importance of different kinds of patterns across different types of stimuli. We thus wondered which methods and constraints more easily and robustly captured underlying firing patterns that are emitted by the same population–though with different combinations and strengths—under many different stimulus conditions. We thus simulated a neural population that responds combining linearly 4 different “ground truth” firing patterns (like above, generated with spatiotemporal Poisson spike trains consisting of periods of strong firing activity for subgroups of neurons, see Fig 3), though in a different way across four different simulated “stimulus sets” (Fig 3). In Fig 3 we report that space-by-time NMF (this was the case also for spatiotemporal NMF and Bayes Poisson Factor, not shown) was able to find the underlying firing patterns robustly across all stimuli sets, whereas the patterns found by orthogonal Tucker-2 were not consistent and reflected the way that these patterns changed across stimuli in the set rather than only the pattern’s firing structure (Fig 3). While having a basis function that easily picks up the changes of patterns across stimuli may be useful for decoding efficiently the stimuli from the neural responses, this property is detrimental for detecting the exact form of these firing patterns. All other methods that applied statistical constraints to the modules shared this inconsistency. We thus conclude that the non-negativity constraint is the most promising to find stereotypical firing patterns that are repeated by the neural population across responses to several types of stimulus conditions.

### Decoding performance of spike train decompositions depending on population firing characteristics in simulated datasets

**Fig 4:**
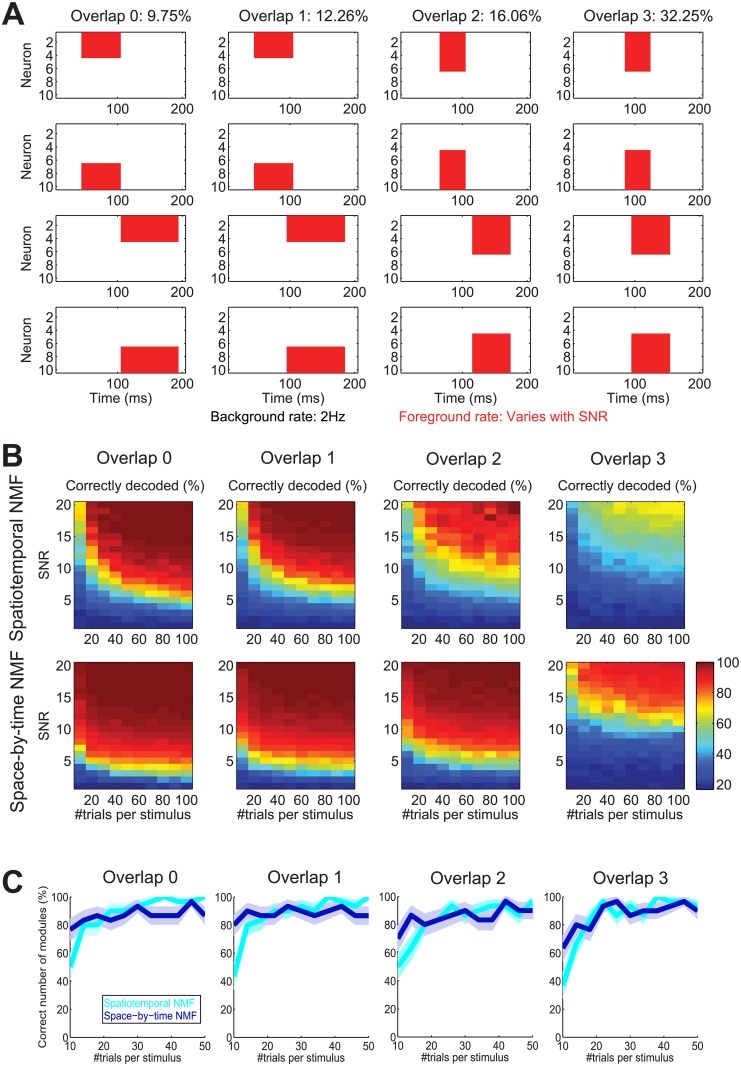
NMF-based stimulus decoding of simulated data. (A) Patterns for spike train generation are plotted with the same conventions as in Fig 2A. Overlap of the patterns is increasing from left to right (overlap 0 to 3). Percentage of this overlap as measured in S1 Text, Section “Quantification of similarity between modules” is plotted at the top. The areas of the patterns are kept constant across overlap levels. (B) Stimulus decoding performance on these simulated data with varying number of trials per stimulus and signal-to-noise ratio (SNR) obtained using spatiotemporal NMF (top row) or space-by-time NMF (bottom row). The background rate was 2 Hz in all simulations. The foreground rate equaled the background rate times the SNR. We used the ground truth numbers of modules (4 spatiotemporal modules for spatiotemporal NMF, 2 temporal and 2 spatial modules for space-by-time NMF) for training and testing the space-by-time NMF algorithm. (C) Percentage of correct selection of the number of modules as a function of the number of trials per stimulus for spatiotemporal NMF (cyan) and space-by-time NMF (blue). We selected the smallest numbers of modules with the maximum test set decoding performance and compared the selected numbers to the ground truth numbers (4 spatiotemporal modules for spatiotemporal NMF, 2 temporal and 2 spatial modules for space-by-time NMF).

To further illustrate the specific advantages of the tensor space-by-time representation over the matrix spatiotemporal one, we next investigated the effectiveness of space-by-time and spatiotemporal NMF in stimulus decoding by constructing simulated spike trains in response to 6 different simulated “stimulus” conditions with different spatiotemporal response patterns. This comparison is fair because both decompositions are subject to the same non-negativity constraint. Again, we generated Poisson spike trains with spatiotemporal patterns consisting of periods of strong firing activity for subgroups of neurons. As in Fig 2, these patterns had the form of four overlapping blocks (Fig 4A). We simulated responses to 6 stimulus conditions, with responses in each condition made up of a combination of two different blocks (S2B Fig). Each block was separable in space and time, meaning that the simulated datasets were fully in line with the space-by-time factorization assumption of space-by-time NMF.

We produced and then analyzed with the spatiotemporal and the space-by-time non-negative decompositions several such simulated datasets in which we systematically varied both the SNR of the blocks (quantified as described above) and the degree of overlap among these simulated patterns. We quantified overlap by means of the geodesic similarity averaged across all module pairs (defined in Eq. (S3) in Supporting Information S1 Text, Section “Quantification of similarity between modules”), and in the simulated dataset we varied this overlap parameter between 9.75% and 32.25%.

In all analyses presented in this section we decoded the presented stimuli from the single-trial coefficients of the NMF decompositions by first computing the NMF modules and training a linear discriminant analysis (LDA) stimulus classifier on a training set (half of the trials) and then using the classifier to decode the stimuli presented on a test set (remaining half of trials) of activation coefficients (c.f. Materials and Methods: Decoding Analysis). We then studied the stimulus decoding performance as a function of the number of trials (total number of training and test set trials) per stimulus, the degree of overlap and the SNR of patterns. We found (Fig 4B) that the SNR of the ground truth patterns present in the data profoundly influenced decoding performance. The larger the SNR of the patterns, the easier it is to decode the stimuli. Decoding stimuli from patterns with relatively low SNR values was far easier with space-by-time NMF than with spatiotemporal NMF (Fig 4B). These results are partly due to the fact that (as demonstrated in Fig 2) space-by-time NMF finds the original patterns in the data even at low SNR values. In this simulation, if the degree of overlap among patterns remains in the range of 16% or below (three leftmost columns in Fig 4A) an SNR of 5–8 is sufficient to achieve perfect decoding of these stimuli even when only few tens of trials per stimulus are available. Increasing the overlap among the firing patterns made it more difficult to decode information. This is because more overlap between the patterns results in more similarity of both the patterns to be decomposed and the responses across stimulus conditions, thus making it harder to both identify the correct modules and decode the stimuli accurately. When the overlap across patterns reaches 32% (rightmost column in Fig 4), an SNR of 15 or more was needed to decode the stimuli with high accuracy. For orthogonal Tucker-2, we found results very similar to those we obtained using space-by-time NMF (S3 Fig).

In all these simulated datasets, decoding performance of space-by-time NMF was at least as good as that of PCA, ICA, FA and Bayes Poisson Factor, and was particularly advantageous for larger overlaps (results not shown). Similar results (in particular, better decoding performance of space-by-time decomposition over the spatiotemporal one especially in challenging SNR and overlap conditions) were found when comparing decompositions with orthogonality constraints (spatiotemporal PCA vs. orthogonal Tucker-2). We emphasize, however, that in this simulation the space-by-time factorization assumption was fully met. We therefore expected that space-by-time methods would outperform spatiotemporal ones.

### Performance of decompositions in correctly retrieving the number of firing patterns in simulated datasets

An arbitrary parameter of all considered decomposition methods is the number of modules (in other words, the dimensionality) chosen to represent the data. In the simulations presented above, we chose the number of modules to equal the number of firing patterns that we embedded in the data. The correct underlying number of modules is, however, unknown in real data and must be determined empirically. Here we propose a principled way to choose, in empirical analysis problems, the number of spatial and temporal modules of the space-by-time NMF, orthogonal Tucker-2 and spatiotemporal modules of the spatiotemporal PCA, ICA, FA and NMF decompositions. For simplicity, we exemplify again the procedure using non-negative decompositions. We select the minimum number of modules that maximize the cross-validated stimulus decoding accuracy (c.f. Materials and Methods, Section “Decoding Analysis”). This procedure should find the most compact representation that preserves all sensory information contained in population activity (see S1 Text, Section “Selection of optimal number of modules” for more details and S4A Fig for an illustration).

We validated this procedure using once more the dataset generating responses to different stimuli with partly overlapping firing patterns plotted in Fig 4A. The percentage of simulated datasets that led to a correct evaluation of the number of modules using this procedure is plotted in Fig 4C as a function of the number of trials per stimulus used to simulate the dataset. The correct number of both space-by-time and spatiotemporal modules was identified more than 80% of the times even down to relatively small data size. Space-by-time NMF reconstructed the number of modules more accurately than spatiotemporal NMF for very low number of trials (10–15). Again, we expected space-by-time NMF to perform better than spatiotemporal NMF, because the space-by-time factorization assumption was true for these simulated data. Similar results were also found for the orthogonal Tucker-2 tensor decomposition (not shown). All in all, these results validate the empirical effectiveness of the decoding maximization procedure for selecting the number of space-by-time modules.

### Responses of populations of retinal ganglion cells in the salamander retina during presentation of natural stimuli

To further probe the ability of the decomposition to extract information-rich patterns of neural population firing, we used multi-electrode arrays to simultaneously record activity of populations of individual retinal ganglion cells (RGCs) in the *axolotl salamander* retina.

In this section, we studied responses obtained when presenting two different classes of natural visual stimuli, which were representative of the general decoding properties of the tensor and matrix decomposition methods on all retinal datasets that were recorded for this paper (see Section “Studying the role of spike timing of retinal ganglion cells in the decoding of image features from population activity” for additional retinal datasets).

The first stimulus set (“Natural Images”) was made of 60 grayscale natural photographs that were flashed on the retina for 200 ms. The second stimulus set (“Natural Movies”) was made of two 60 s long natural movie clips presented with a 30 Hz frame rate. Both movies (whose temporal correlation properties are reported in S5 Fig) contained a wealth of different types of motion within complex visual sceneries, including global image shifts and drifts as well as local object movement. We recorded n = 38 and n = 49 cells from two retinas in response to both stimulus sets.

**Fig 5:**
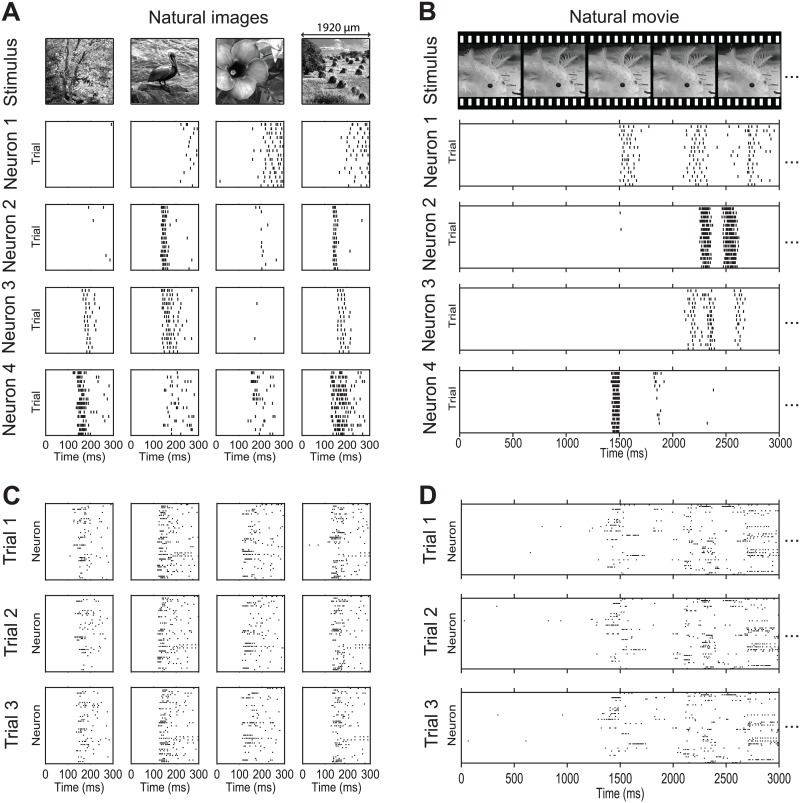
Examples of RGC spike raster plots in response to natural stimuli. (A) Raster plots of the spike times of four representative neurons in response to four example natural image stimuli over a 0–300 ms post-stimulus period. Each column shows the responses to the image shown at the top. Each row of raster plots shows responses from the same neuron. The scale bar above the image represents the width of the projected image on the retina. (B) Raster plots in response to a 3 s section of a salamander movie stimulus. For space reasons, only five consecutive example frames from the movie are shown at the top for illustration purpose. (C, D) Like A (for image stimuli) and B (for a movie stimulus), but each raster plot shows the responses of the whole population on a single trial. Each plot corresponds to one trial.

Example responses of four neurons to both sets of natural stimuli are reported in Fig 5. Responses of individual neurons to images (Fig 5A) were consistent across trials with latency in the range 70–150 ms. Single neurons showed a reliable and cell-specific modulation by the images of both the total number of spikes elicited and the latency and duration of neural responses, suggesting that in principle both spike counts and spike timing of individual neurons may contribute to population coding of these images.

Responses to movies (Fig 5B) showed phasic elevation of firing at specific points during the movie. The firing rate increases were cell-specific and highly repeatable across trials. This indicates that determining which part of the movie (or “scene”) was being presented can be decoded from population activity taking into account both the spatial and temporal dimension of the population code.

Single-trial population activity (Fig 5C and 5D) in response to both movies and images showed the presence of repeatable patterns of simultaneous firing across several neurons at different times, suggesting that space-by-time factorizations may provide a good way to describe these population firing patterns.

### The spatial and temporal modules obtained from simultaneously recorded populations of retinal ganglion cells

We then applied all considered methods to decode the visual information carried by these responses. For the following analyses, we binned the single-trial spike trains with a 10 ms resolution (see below for a discussion of how finer temporal binning affected the results). We analyzed a dataset of 30 trials of each natural image and each movie type, and (unless otherwise stated) we randomly separated these 30 trials into 15 training trials (used to compute the decomposition modules and to train the decoder) and 15 test trials (used to compute decoding performance).

Before considering how the spatial and temporal modules provided by the decompositions may be used to decode visual stimuli, it is worth considering the shape of the spatial and temporal modules themselves. Since they were selected to maximize visual information with the smallest number of parameters, these modules can be interpreted as indicating the spatial and temporal resolution at which the neural population code should be read out.

**Fig 6:**
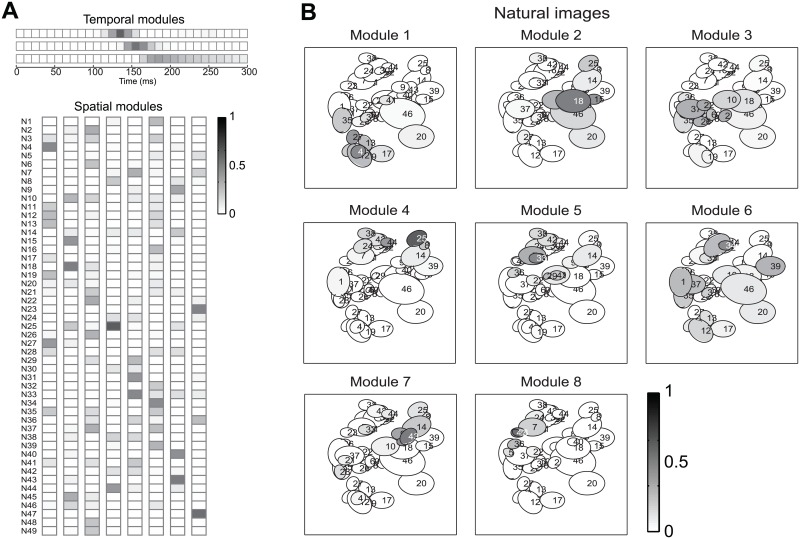
Space-by-time NMF decomposition of responses of populations of retinal ganglion cells to natural images. Example of the space-by-time NMF decomposition of a dataset comprising the single-trial population responses of 49 RGCs to a set of 60 natural images. (A) The three temporal modules (top) and the eight spatial modules (bottom) obtained from the training set of these data. (B) The eight spatial modules plotted over the receptive fields of the respective neurons (each ellipse shows the receptive field of a recorded retinal ganglion cell).

**Fig 7:**
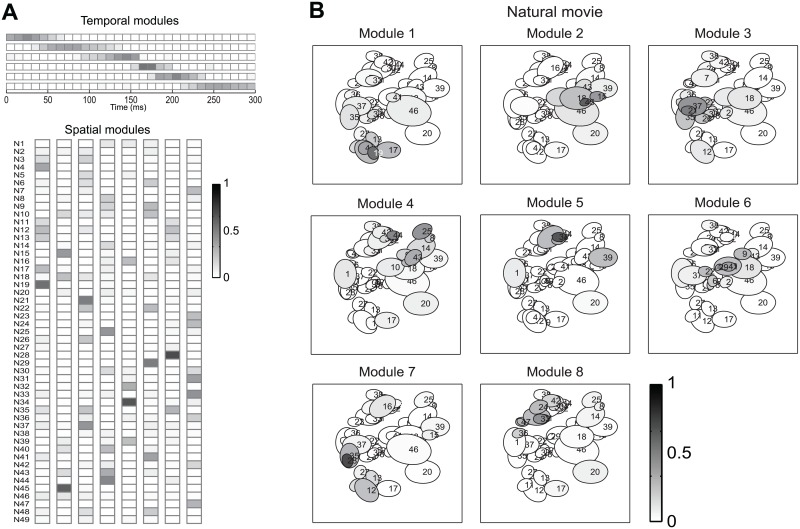
Space-by-time NMF decomposition of responses of populations of retinal ganglion cells to natural movies. Example of the space-by-time NMF decomposition of a dataset comprising the single-trial population responses of 49 RGCs to a 60 s natural movie. (A) The six temporal modules (top) and eight spatial modules (bottom) obtained from the training set of these data. (B) The eight spatial modules plotted over the receptive fields of the respective neurons (conventions as in Fig 6B).

We first considered the modules obtained with space-by-time NMF. Fig 6A shows such temporal and spatial NMF modules obtained from responses of retinal ganglion cells in the training set of an example session (with n = 49 simultaneously recorded cells) when presenting the images. Each spatial module describes the relative degree of simultaneous firing of groups of neurons. Large amplitudes of a set of cells within a module indicate that those cells tend strongly to fire together. We visualized the spatial modules both as vertical vectors (Fig 6A) of activation across cells (to match their representation in Fig 1), and also by plotting their amplitudes as gray values within the receptive fields of the cells (Fig 6B). This latter plot revealed that spatial modules appear to be sparse (with only a fraction of neurons strongly active in a given module) and also retinotopically clustered: neurons that belong to the same module tend to have spatially neighboring receptive fields (RFs, c.f. Materials and Methods, Section “Multielectrode recordings from retinal ganglion cells”), likely because neighboring cells receive more conjunct stimulation by the natural stimuli and thus fire together more often than distant cells do. Importantly, the space-by-time NMF decomposition was compact: it only needed a small number of spatial modules (eight) to capture all information about the 60 experimental stimuli in these data. Space-by-time NMF also yielded very similar spatial firing patterns when applied to responses to natural movies collected from the same population of cells (Fig 7), highlighting the ability of non-negative decompositions to find firing patterns that occur robustly across stimuli. For comparison, we also applied to the natural image RGC responses a space-only decomposition of NMF which yielded modules that were much more similar to those of space-by-time NMF (S6D Fig), validating the power of the non-negative constraint to uncover such interpretable and sparse spatial firing patterns.

To gain an intuition about the spatial properties of the retrieved basis functions when using decompositions that impose statistical constraints on the modules and to compare them with those obtained in Fig 6 with a non-negativity constraint, we extracted spatial modules from the same dataset using the orthogonal Tucker-2 decomposition. To retrieve as much information as possible from neural responses, the orthogonal Tucker-2 required 16 (rather than 8 as in the NMF) spatial modules, thus being a less compact representation when compared to space-by-time NMF. Moreover, the modules were much less spatially compact and took both positive and negative values (S7 Fig, Panel B). This held even when imposing the orthogonal Tucker-2 to have the same number of modules (eight) as those needed by the space-by-time NMF to extract its maximal stimulus information (S7A Fig). For further comparison, we also computed from the same dataset spatial PCA, ICA, FA and NMF modules by a space-only decomposition, i.e. we factorized the binned neural responses into trial-independent spatial modules and (module-by-time) trial-dependent activation coefficients (c.f. Materials and Methods, Section “Space-only decomposition models”). The spatial modules obtained using PCA, ICA and FA on the example responses to natural images were much less spatially compact and took both positive and negative values (S6 Fig, panels A-C), whereas the spatial NMF modules were very similar to those obtained using space-by-time NMF (S6 Fig, panel D). In conclusion, spatial modules obtained imposing constraints on their statistical relationships seem less directly interpretable than NMF in terms of patterns of simultaneous neural activation.

The temporal modules found with space-by-time NMF for the same Natural Images example session are reported in Fig 6A. (Responses were considered in the 0–300 ms post-stimulus window, as it contained the entire response to the flashed image but excluded the off response). Two of these temporal modules had a non-zero amplitude only in a short time region (50 ms wide) centered around two different times (140 and 160 ms post-stimulus onset, respectively) shortly after the onset of neural response to the images. These two temporal modules thus described the difference in neuronal response latencies across cells and stimuli. A third module had non-zero amplitude over a wide 140 ms interval starting at approximately 160 ms post-stimulus and described the sustained part of the neural response. Similar time decompositions for natural images were obtained for the other session (not shown). This suggests that in order to describe the retinal responses to flashed natural images (and then extract the visual information that these neurons carry) it is sufficient to track with few-tens-of-ms precision the latency of the early response and then count spikes over hundred or so ms in the sustained response. The temporal modules obtained with orthogonal Tucker-2 with the same dataset were less localized in time and were both positive and negative. Also, the number of modules was higher (4 modules, rather than 3, were needed by orthogonal Tucker-2 to retrieve the most stimulus information), yielding a less compact representation than space-by-time NMF. This again underlines the advantage of the non-negativity constraints in extracting compact recurring spatial firing patterns expressed by the microcircuitry.

The temporal modules obtained for the movie dataset partitioned the movie scenes into time segments that span their range, effectively allowing to keep track of firing rate changes at the tens-of-ms scale within a scene, and thus potentially tracking dynamic variations in the properties of visual features across frames that could be used to gain information about which scene was being presented (see S5 Fig for the time scale of time variations of some movie features).

An arbitrary parameter of the analysis is the size of the temporal window used to bin the spikes over time, which was set to 10 ms in the previous analyses. We verified that we could use this specific window size without loss of information or generality. We found (both with space-by-time NMF and orthogonal Tucker-2, the two best performing methods for stimulus information decoding, see next Section) that binning the spike trains at a finer temporal resolution did not increase the amount of visual information that we could extract and did not change the temporal modules. In particular, it did not lead to temporal modules with a finer temporal structure than those reported in Figs 6 and 7. This suggests that visual information carried by these populations can only be decoded by registering spike trains with a time resolution of the order of 10 ms (see also S1 Text, Section “Effective temporal precision”).

### Decoding naturalistic visual information from simultaneously recorded populations of retinal ganglion cells

To gain a more quantitative understanding of how various decompositions describe the visual information content of population activity, we compared how well we could decode visual information using the single-trial activation coefficients obtained with each considered method. We used an LDA decoder, a 50%-50% cross-validation and we chose for all methods the number of modules that maximized decoding performance.

**Fig 8:**
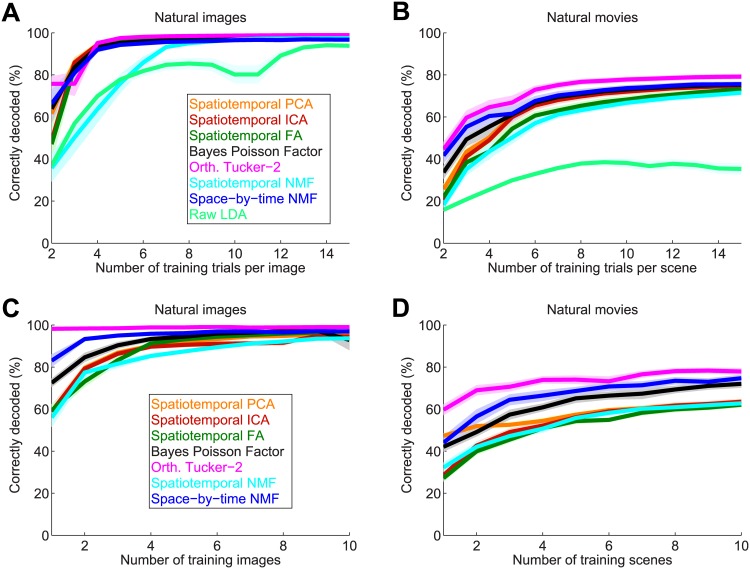
Performance in decoding natural visual stimuli using various decompositions of single-trial neural population activity. Decoding accuracy (% of correct decoding) is shown for spatiotemporal PCA (orange), ICA (red), FA (dark green), Bayes Poisson Factor (black), orthogonal Tucker-2 (magenta), spatiotemporal NMF (cyan) and space-by-time NMF (blue). (A, B) Decoding accuracy as a function of the number of training trials per stimulus averaged over all image datasets (A) or all movie datasets (B). The light green lines show the performance of LDA applied to the binned population spike trains. (C, D) Robustness of the methods with respect to the number of training stimuli. The number of stimuli that were used for training the components/modules is varied on the x-axis and averaged over all image datasets (C) or all movie datasets (D). Performance was evaluated on different stimuli. Lines and shaded areas indicate mean and SEM over all recordings sessions, respectively. In all panels, chance level is at 1/60 = 1.67%.

We first computed decoding performance when varying the number of trials per stimulus in the training set. Fig 8A and 8B show the results averaged over all image and movie test datasets, respectively. For all datasets and methods, decoding performance increased with the number of training trials. On both datasets, raw LDA performed significantly worse than the other methods, most likely due to the strong overfitting of the training set resulting from the large number of parameters used by this method. On the image datasets, spatiotemporal PCA, ICA, FA, orthogonal Tucker-2 and space-by-time NMF performed all very similarly and extremely well, reaching close to 100% even with as few as 4 trials per stimulus, thus demonstrating their effectiveness in capturing all visual information with a small set of modules. On the movie test datasets, the three tensor methods (orthogonal Tucker-2, space-by-time NMF, Bayes Poisson Factor) performed significantly better than spatiotemporal NMF, PCA, ICA and FA (Wilcoxon rank sum test; *p* < 0.01), particularly so for lower number of training trials. This highlights that the tensor decomposition assumption is the most crucial one for robust and highly efficient decoding performance. Among the tensor methods, the orthogonal Tucker-2 was the best one (Wilcoxon rank sum test; *p* < 0.01), followed by space-by-time NMF and then by the Bayes Poisson Factor. This highlights the power of the orthogonality constraint for information extraction which is apparent also in the spatiotemporal case, in which spatiotemporal PCA outperforms spatiotemporal NMF.

In real data we cannot compute with certainty the total amount of sensory information carried by neural population activity. However, in previous simulations (Fig 4) we investigated the range of SNR, number of trials per stimulus and degree of overlap among patterns under which the NMF recovers the entire information in the neural responses. To compare our data with these, we measured the SNR and overlap parameters in RGCs. We found that space-by-time NMF modules obtained from the RGCs had an SNR between 74 and 621 and an overlap between 0.16% and 3.75% (see S1 Text, Section “Measure of SNR and overlap of modules in real retinal data” for details). Given that we showed above that for a sufficient number of trials tensor methods such as space-by-time NMF perform very well for much lower SNR and much larger overlaps than those measured in these real data, we expect that the results obtained with ten or more training images captures a large fraction of the total information content of the analyzed RGC population.

We then tested how well the decomposition methods generalize to neural population responses to stimuli that were not used to train these methods. This further test is important because it may give us hints to understand if the methods can identify stereotypical firing patterns that are determined by the hard wiring of the neural circuitry and so are emitted (though in different combinations) through many stimulus conditions. We randomly separated stimuli into a set A with 50 stimuli that were not used for training the modules but only for testing, and a set B with 10 stimuli from which training stimuli were drawn. We then varied the number of stimuli that were drawn from set B for training the components/modules, while fixing the number of training trials per stimulus to 15. The decoding performance obtained from this analysis on the test set for image and movies are shown in Fig 8C and 8D, respectively. In both cases, decoding performance of all methods increases with increasing number of training stimuli. However, tensor space-by-time methods performed significantly better than the spatiotemporal matrix methods on both datasets (Wilcoxon rank sum test; *p* < 0.01), suggesting again that the space-by-time factorization assumption appears to be essential for robust and efficient decoding performance. In particular orthogonal Tucker-2 and space-by-time NMF gave excellent decoding performance on the test set even when using 2–4 stimuli for training, with orthogonal Tucker-2 performing significantly better than space-by-time NMF on both datasets (Wilcoxon rank sum test; *p* < 0.01), again highlighting the effectiveness of the orthogonality constraint for information extraction.

The excellent generalization properties of space-by-time NMF and orthogonal Tucker-2 prompted a closer look at the stability of their modules as a function of the number of trials used for training the modules. We thus quantified for these two tensor methods (S8 Fig) the module recovery similarity quantified as the geodesic similarity between the modules recovered for the full number of trials per stimulus and the modules recovered for a lower number of trials per stimulus (c.f. S1 Text, Section “Quantification of similarity between modules”). In all cases, we found that the space-by-time NMF modules were more consistent (greater module recovery similarity) than those obtained with orthogonal Tucker-2. For the temporal modules, this difference was not significant on neither the image datasets, nor the movie datasets (S8A and S8C Fig, Wilcoxon rank sum test, *p* = 0.07 and *p* = 0.15, respectively). For the spatial modules, however, the difference was quantitatively large on both the image datasets and the movie datasets (S8B and S8D Fig, Wilcoxon rank sum test, *p* < 0.001). These results show the superiority of space-by-time NMF compared to orthogonal Tucker-2 in detecting consistent and interpretable patterns from real data recorded from RGCs in response to natural stimuli.

To further show the module detection robustness of space-by-time NMF modules, S9A Fig shows representative variability of temporal and spatial modules for one experiment with natural images. For identifying these modules, we drew 1, 5, or 10 distinct training stimuli from the total set of 60 images and applied space-by-time NMF to the set consisting of all training trials of the drawn images. The resulting identified spatial and temporal modules were remarkably consistent, indicating again that space-by-time NMF modules generalize well across stimuli.

An interpretation of these results, consistent with our earlier findings on simulated data (Fig 3), is that space-by-time NMF is better at finding the stereotyped patterns of firing in the data (and thus can give a highly robust similarity of modules across stimuli), whereas orthogonal Tucker-2 is better at finding how these patterns vary across stimuli sets (and thus can give a highly robust decoding of information).

### Quantifying the relative importance of spike timing and single-neuron firing rates using space-by-time tensor decompositions with a permutation procedure for the decoding of visual information from the retina

Having demonstrated the power and plausibility of tensor decompositions of single-trial population spike trains, in the following we concentrate on investigating in more detail how they can be used to understand neural population coding. In particular, we focus on space-by-time NMF (which for the full dataset of RGC gave as good a decoding performance as the orthogonal Tucker-2 but had more interpretable modules) to investigate the relative importance of space (i.e. the neuron-to-neuron differences in response properties) and time (i.e. temporal structure of neural activity) for neural population coding of sensory stimuli.

The total information extracted with space-by-time NMF from population spike trains contains both the information carried by the space and the time dimension of neural activity. In the following we will thus call the information obtained from the space-by-time NMF the “space-and-time information”. In what follows, we quantified how much of this information can be attributed to space and time, using a permutation procedure (c.f. S10 Fig).

To quantify the specific contribution of space to the total information, we first randomly permuted each single-trial population response along the time dimension (without permuting responses along space), and then performed space-by-time NMF and decoding. The so-obtained data lost all information in the neural response timing and only contain information carried by the stimulus-to-stimulus and neuron-to-neuron differences in firing rates. We thus refer to this procedure as using “space-only information”. The difference between space-and-time and space-only information quantifies how much of the total information can only be retrieved using the time dimension.

To determine the specific contribution of time to the total information, we first randomly permuted single-trial population responses along the space dimension (without permuting responses along time), and then performed space-by-time NMF and decoding. The so-obtained data lost all information in the neuron-to-neuron response differences and only contain information carried by the temporal structure of the pooled neural activity. We thus refer to this procedure as using “time-only information”. The difference between space-and-time and time-only information quantifies how much of the total information can only be retrieved using the space dimension.

The ability of this permutation procedure in identifying the relative importance of space and time in the population code is illustrated in S10 Fig, and S1 Text, Section “Permutation procedure to identify the relative importance of space and time”, where we generated simulated spike patterns that either had all information in the space dimension, all information in the time dimension, or information genuinely present in both space and time. We found that the permutation procedure was able to differentiate correctly among those alternatives.

### Studying the role of spike timing of retinal ganglion cells in the decoding of image features

To investigate retinal population coding of image features, we used the space-by-time NMF to analyze population responses to various sets of static images flashed onto the retina for 200 ms. Applying flashed images is an experimental paradigm often used to assess how the retina processes new visual information for an image that suddenly comes into focus, for example, following a saccade or a head movement [12]. In all these analyses, we decoded which image from a given set was presented by assessing the single-trial NMF activation coefficients obtained when considering space-and-time, space-only or time-only information. We used all image stimuli in the set for training and divided responses to each stimulus 50%-50% into training and test trials.

We first considered decoding of the set of 60 different “natural images” (S11 Fig), whose NMF decomposition of the associated neural responses was presented above. In this natural image set, using space and time information led to an almost perfect image decoding (percent of correct decoding of 98% ± 0.5%). Using time-only information led to a very large loss of decoding performance (a decrease of 76% ± 0.8% from the space-and-time information case, *p* < 0.001, two-tailed t-test), highlighting the all-important contribution of neuron-to-neuron response differences to this neural population code. Conversely, using space-only information led only to a very small but significant decrease of decoding performance (2% ± 0.3% decrease, *p* < 0.05, two-tailed t-test). This finding suggests that nearly all information about natural images in this population code can be extracted simply from the firing rates of the neurons, without considering the temporal structure of firing. However, this finding may either mean that there is no information in spike timing (and thus vision of these images must rely on spike rates) or that information about images is redundantly encoded in both rates and spike times. To disambiguate between these scenarios, we performed two analyses.

First, we investigated whether the fact that spike timing could only add little information was partly a result of a “ceiling effect”: Given that spike counts of individual neurons (“space-only code”) by themselves carried enough information for nearly perfect discrimination, no other neural code can add much to it. We performed a control analysis where we reduced the population size (S12 Fig) and thereby degenerated the decoding accuracy of the space-and-time code, and we indeed found that in such case the additional information carried by time was larger (loss of information of the space-only code was up to 23% for 1/8^th^ population size), compatible, with the presence of some “ceiling effect” (See S12 Fig and S1 Text, Section “Ceiling effect in natural image datasets” for full details).

**Fig 9:**
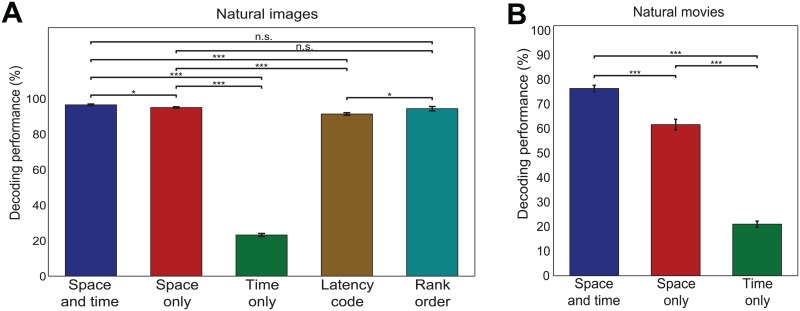
Quantification of importance of space and time to decoding performance. Comparison of decoding performance after training on unshuffled responses (space-and-time), after shuffling bins across time (space-only), after shuffling bins across neurons (time-only), after keeping only the first spike of each neuron in each trial (latency-code, panel A only), and for the rank order decoder (rank order, panel A only). (A) Results averaged over all image datasets. (B) Results averaged over all movie datasets. *p<0.05; ***p<0.001; two-tailed t-test. Error bars indicate s.e.m.

The second possibility is that a timing code carries information, but that this information is almost fully redundant to that carried by firing rates only. To understand whether timing itself carried information, we computed the information conveyed by the first-spike latency of the discharge of each neuron in response to the images. To do so, we decoded the spike trains with a space-by-time NMF performed exactly as above but applied to spike trains in which we deleted all spikes apart from the first one for each neuron and for each trial. This information, which we called latency-code information, is a lower bound to the information carried by the whole spike train, because timing of later spikes can only add information [16]. We found (Fig 9A) that the latency-code decoding performance was very close to the space-and-time and space-only performances, indicating that almost all information is indeed redundantly carried by both neuron identities and first-spike latencies (5% ± 0.3% drop compared to the space-and-time information, *p* < 0.001, two-tailed t-test).

As a further test of the importance of latency for information coding, we also decoded the first-spike latency information using a state-of-the-art rank-order decoder (c.f. Materials and Methods: Section “Decoding analysis”) which evaluates relative differences of first spike latencies in the population [65–68]. Rank-order decoding led to a slightly higher decoding performance than that obtained with latency-code space-by-time NMF (94% vs. 91%, *p* < 0.05), suggesting that our approach is competitive with state-of-the-art latency decoders. More importantly, this also means that information in first-spike latencies could be decoded by a downstream neural system that does not have independent knowledge of the stimulus time.

The large variety in the set of natural images (S11 Fig) whose responses were analyzed above leads typically to large image-to-image differences in RF stimulation. It is possible that total spike counts over the entire response period are sufficient to discriminate among these coarse image differences, whereas precise spike timing is needed to discriminate finer image differences. To test this hypothesis, we applied space-by-time NMF to a set of RGCs (n = 54) simultaneously recorded in response to a set of 60 full-field grating stimuli (again flashed for 200 ms). The gratings had a bar size slightly larger than typical RGC receptive fields, and they differed from each other only by a spatial shift much smaller than the typical RF size (see Material and Methods, Section “Stimulation with flashed natural images and gratings”). Thus, discriminating among these images from neural responses requires discriminating responses to fine image differences within the RF. Further, this kind of artificial image set had been shown to exhibit strong timing-dependent information in RGC responses in the form of first-spike latencies and relative spike timing [12], and it is thus a good test of the capability of the space-by-time NMF to detect spike timing structure.

**Fig 10:**
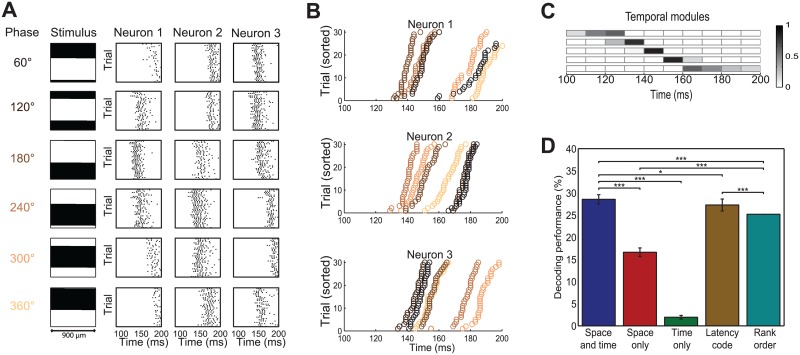
Analysis of responses to flashed gratings. (A) Examples of RGC spike raster plots in response to 6 of the total of 60 flashed gratings. Each row shows the responses to the same grating which is shown on the left. Each column of raster plots shows responses from the same neuron. For better illustration of timing relations we show only the time range from 100 ms to 200 ms after stimulus onset, but analyzed the range from 0 ms to 300 ms. The scale bar below the grating represents the width of the projected image on the retina. (B) First spike latencies of three representative neurons, sorted for each neuron individually. Each color shows the latencies for a different grating phase. (C) Five temporal modules that were identified by space-by-time NMF. Different rows correspond to different modules. (D) Comparison of decoding performance on unshuffled responses with space-by-time NMF (denoted as space-and-time), after shuffling bins across time (space-only), after shuffling bins across neurons (time-only), after keeping only the first spike of each neuron in each trial (latency-code) and for the rank order decoder (rank order).

Fig 10A shows responses of three representative RGCs to flashed gratings of different spatial phases, which clearly show that the first-spike latencies of these responses depend on the spatial phase. This phase-dependence of latencies is revealed even more clearly in Fig 10B, in which first-spike latencies of these neurons are shown in different colors for different grating phases. Fig 10C shows the temporal modules that we obtained by applying space-by-time NMF to this dataset. We found a clear relation between these modules and the spike latencies that are shown in Fig 10B: the time at which the temporal modules peak align very well with first spike latencies of the neurons, suggesting that these modules describe latency differences and can be used to describe differences in responses to different stimuli.

We then applied spatiotemporal NMF, space-by-time NMF and the permutation procedure (S10 Fig) in order to understand the relative importance of space and time in the population coding of spatial phase of images. First, we compared decoding performance of spatiotemporal NMF and space-by-time NMF. We did not find a significant difference between these two procedures (*p* = 0.1, two-tailed t-test), thus showing no evidence for a role of non-factorizable contributions in encoding these flashed gratings. Consistent with the visual inspection of the responses, the permutation analysis showed a strong decrease of information (decoding performance drop of 42% ± 1.37%, *p* < 0.001, Fig 10D) in the space-only code in which spike timing was destroyed. This means that the retinal population code of spatial phase contains unique information in spike timing that cannot be recovered from total spike counts alone. Visual inspection of responses, as well as previous analysis of small populations [12] suggests that first-spike latencies are a key component of this population code. To test this hypothesis at the larger population level of tens of cells, we first erased all spikes but the first from each neuron in each trial and we then recomputed the decoding performance subjecting this first-spike-only dataset to the space-by-time-analysis. We found that first-spike latencies carried almost all information contained in the full spike trains (27% vs. 29%, *p* < 0.05), demonstrating that the information carried by later spikes about image spatial phase is redundant to that already carried by first spikes. We confirmed these results with the orthogonal Tucker-2 method, obtaining qualitatively similar results (S13A Fig). Decoding the first-spike latency information using a rank-order decoder led to a slightly lower decoding performance than that obtained with space-by-time NMF (25% vs. 27%, *p* < 0.001), corroborating the effectiveness of our approach compared to current methods and suggesting that, using our approach, knowledge of the stimulus time is not required to decode information in first-spike latencies.

**Fig 11:**
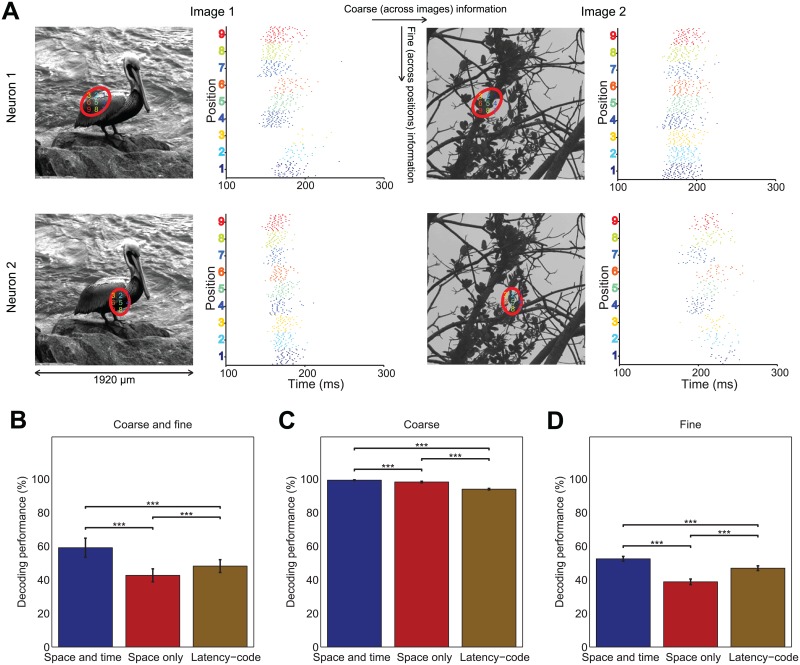
Analysis of responses to shifted natural images. (A) Examples of RGC spike raster plots in response to shifted natural images. The top row shows the receptive field and raster plots of representative neuron 1. The bottom row shows the same for neuron 2. The two columns to the left are for image 1 and the two columns to the right are for image 2. The scale bar below the image represents the width of the projected image on the retina. The red ellipses indicate the receptive field of the neuron in the presented image. The numbers in the ellipse denote the directions from the center of the ellipse in which the image was shifted. Raster plots show the spike times of the neurons in response to the natural image stimuli in the 9 indicated positions. For better illustration of timing relations we show only the time range from 100 ms to 300 ms after stimulus onset, but analyzed the range from 0 ms to 300 ms. Each row of raster plots shows responses from the same trial. (B-D) Comparison of decoding performance on unshuffled responses with space-by-time NMF (space-and-time), after shuffling bins across time (space-only), and after keeping only the first spike of each neuron in each trial (latency-code). (B) Performance of decoding image id and image position. (C) Performance of decoding image id for each position, averaged over positions. (D) Performance of decoding image position for each image id, averaged over images. ***p<0.001; two-tailed t-test. Error bars indicate s.e.m.

The importance of spike timing for coding small image differences was tested in the above and (to our knowledge) in previous experiments only using artificial grating stimuli. To verify the hypothesis that the retinal population spike times carry information about fine-scale features of natural images that cannot be recovered from total spike counts, we performed a new experiment in which we simultaneously recorded RGC responses (two experiments, n = 23 and n = 37 cells) in response to a set of 81 natural images that contain both coarse and fine differences in within-RF image features. This “shifted natural image set” was constructed by first taking 9 different natural photographs, and then presenting 9 different versions of each photograph obtained by spatially shifting each of them by an amount much smaller than the typical RF size (see Fig 11A).

We first decoded which of these 81 stimuli had been presented. Decoding this set, which involves discrimination both between coarse image features (differences across different images) and fine image features (differences between shifts of the same image), appeared to require timing information. In fact, we found (Fig 11B) that destroying information in the time dimension led to a large loss of decoding performance (space-only decoding was 16% ± 2.85% less accurate than space-by-time decoding, *p* < 0.001). We then identified the differential contribution of spike times and spike rates to coarse and fine image coding. We first considered a set of 9 stimuli, obtained by grouping all 9 shifts of an image into a single stimulus class whose decoding required discriminating only coarse image differences (i.e. discriminating which of the different images was presented). Visual inspection of the responses indicated that the total spike counts differed between coarse images. Indeed, we found that this “coarse image decoding” could well be performed based on total spike counts alone, without considering spike times: space-only decoding was only 1% ± 0.59% less accurate than space-by-time decoding, *p* < 0.001 (Fig 11C). We then considered another set of 9 stimuli, obtained by considering only the 9 shifts of a single image whose decoding required discriminating only fine image differences (i.e. discriminating which of the different small shifts of the same image was presented). In this case, and in agreement with the grating results, we found (Fig 11D) that this “fine image decoding” required spike timing: neglecting spike times led to a large loss of decoding performance (space-only decoding was 14% ± 1.84% less accurate than space-by-time decoding, *p* < 0.001). Notably, for all considered types of image discrimination the latency code (again evaluated with space-by-time NMF) carried a large fraction of the total information, and latency code information was larger than spike count information for all stimuli sets whose decoding needed fine discrimination (Fig 11). Again, we confirmed these findings with the orthogonal Tucker-2 method, obtaining qualitatively similar results (S13B–S13D Fig).

Decoding performance depending only on coarse image features (Fig 11C) significantly increased when using spatiotemporal NMF instead of space-by-time NMF (*p* < 0.001, two-tailed t-test). However, this increase was small (0.66%). Decoding performance depending on both coarse and fine image features (Fig 11B) as well as decoding performance depending only on fine image features (Fig 11D) decreased significantly when using spatiotemporal NMF instead of space-by-time NMF (*p* = 0.011 and *p* < 0.001 respectively, two-tailed t-test). This decrease might be attributed to the small number of trials of this specific condition in the dataset (for which only 19 trials per stimulus were available) and with the fact that matrix spatiotemporal NMF is more data-hungry than space-by-time NMF (Fig 4).

While shuffling in either space or time also increased the reconstruction error (results not shown), the consistency of information values obtained by different methods (Figs 10D and 11, S13 Fig), and the saturation of decoding performance for the presented number of modules (although reconstruction error further increased with the number of modules for the shuffled datasets) suggest that the observed information drop is due to loss of intrinsic space or time information in the data rather than to reconstruction failure.

All in all these results confirm our hypothesis that RGC responses carry unique information about fine image differences in spike timing also in a natural image context, whereas information about coarse image differences is also carried in total spike counts. These findings highlight—at the population level and for both artificial and natural images—the importance of retinal spike timing and in particular of first-spike latency for coding fine spatial differences in image features.

### Investigating the temporal precision of image coding in the retina

The above results show that spike timing carries information about fine image features that cannot be possibly recovered from total spike counts over the entire response window. An important question concerns the temporal precision at which spike timing information is carried. To address this question, we evaluated decoding performance at various effective temporal precisions for the flashed gratings dataset and the shifted natural images dataset. To vary the effective precision, we followed the procedure outlined in [24]. We started from a precision of 10 ms (corresponding to the time bin size we used) because we found that performing decompositions with more finely binned data did not increase information. We then compared the decoding performance when spike times were recorded with this 10 ms precision to that obtained after shuffling 2 consecutive bins (20 ms), 5 consecutive bins (50 ms), 10 consecutive bins (100 ms) and 30 consecutive bins (300 ms).

For the flashed gratings dataset (S14A Fig), reducing the effective precision from 10 ms to 20 ms, 50 ms, 100 ms and 300 ms led to highly significant performance drops of 10.9%, 30.6%, 40.7% and 41.8% relative to the 10 ms precision (*p* < 0.001 for all comparisons, two-tailed t-test). For the shifted natural image datasets, reducing the effective precision from 10 ms to 20 ms did not significantly decrease decoding performance that depended on coarse and fine image features (S14B Fig, *p* = 0.34, two-tailed t-test) or coarse image features (S14C Fig, *p* = 0.58, two-tailed t-test) but did significantly increase decoding performance that depended on fine image features (S14D Fig, *p* = 0.03, two-tailed t-test). Decreasing temporal precision to more than 20 ms led to highly significant decreases in decoding performance for all considered types of image discrimination and ranging between 21.6% and 27.8% relative to 10 ms performance for coarse and fine features, between 24.9% and 26.1% for fine image features, and between 1.1% and 1.9% for coarse image features.

In summary, these results show that coarse image decoding could well be performed based on rates only, whereas fine image decoding required spike timing measured on a 10 ms scale. The importance of 10 ms scale spike times and in particular spike latencies for information decoding suggests that temporal variations of firing rates (especially in the early part of the response) are an important component of the retinal population code for natural images.

### Studying the role of spike timing of retinal ganglion cells in the decoding of time-varying visual stimuli

We finally considered results obtained in response to natural movies. Unlike the flashed image stimuli, these stimuli contain both image feature information and dynamic information about the evolution of these features over time. Here, using space-and-time information led again to a very good (though not close to perfect) decoding of stimulus identity (percent correct scene decoding 76% ± 1.3%). Similar to our previous results with images, there was a very large loss of decoding performance when using time-only information (a decrease of 73% ± 1.4% from the space-and-time information case, *p* < 0.001), confirming the important contribution to this code of neuron-to-neuron response differences for movies. Importantly, using space-only information with movies led to a large loss of decoding performance (a decrease of 20% ± 1.7%, *p* < 0.001) compared to the space-and-time information case. This means that spike timing carries information about natural movies that cannot be recovered by the time-averaged firing rates only. We did not repeat the first spike latencies analyses for the movie datasets because selection of scenes is arbitrary and so a latency-based analysis relative to arbitrary scene onsets would not be of neurophysiological interest.

## Discussion

We investigated tensor-based methods that decompose single-trial population spike trains into a combination of trial-independent spatial firing patterns (describing which combinations of neurons fire together), temporal firing patterns (describing the temporal activation profiles of the spatial patterns) and trial-dependent coefficients (describing the strength by which each combination of a spatial and temporal pattern is recruited in a given trial). We evaluated the effectiveness of these decompositions in both extracting efficiently the sensory information carried by neural populations along both space and time dimensions, and identify the firing patterns present in the population spike trains. The implications of this spike-train analysis methodology and its application to population coding by retinal ganglion cells are discussed in the following.

### Factorization of space and time in neural activity

One of our main findings was the high decoding performance, data robustness and generalization properties of tensor decompositions assuming that the space and time dimensions of neural population codes can be separately factorized. The work presented here can thus contribute to establishing space-by-time decompositions as a mainstream approach in spike train analysis as it happened for many other fields [69–73].

From the neurophysiological point of view, the success of space-by-time tensor decompositions in describing and decoding retinal responses suggests that, while surely not all population firing patterns are exactly separable, many patterns of firing can be approximately described in a factorized way as sequential temporal activations of spatial groups of neurons that fire together (an observation also made in [74]). The robustness advantages of the space-by-time decompositions seem to overcome the possible imprecisions of this approximation, suggesting that in general there is a lot more to be learned by factorizing neural population responses in space and time.

For non-negative decompositions in particular, we note that, while NMF is, in principle, naturally suited to study neural population coding, its applications to spike train analysis have been limited to a few cases [74–76] where the spatiotemporal version of NMF was applied. A reason that may have prevented NMF to become a popular spike train tool so far is that, as we showed here, the direct application of spatiotemporal NMF to single-trial spike trains seems not data robust. However, our result that space-by-time separability enormously increased the ability of NMF to extract single-trial patterns even in conditions of lower signal-to-noise ratio and higher firing pattern overlap may contribute to a wider use of NMF in population spike train analysis.

### What space-by-time decompositions add to existing methodologies for studying population coding

Many previous methods performed a spatial dimensionality reduction (with techniques such as PCA [77, 78], ICA [32, 33] and FA [79]) of population spike trains, describing the distribution across neurons of simultaneous patterns of firing with a small number of spatial modes. These techniques provided important insights into the structure of population activity, but did not aim at obtaining low-dimensional representations of the temporal structure of firing of cell assemblies. Our space-by-time method instead provides a low-dimensional decomposition of population codes simultaneously performed along both space and time dimensions, and is suitable to describe compactly both the relative pattern of simultaneous firing across neurons and the sequential activation over time of this firing. This, as we showed in our analysis of RGCs, is useful to study how information is distributed among cells and across time.

One increasingly popular set of tools to analyze population recordings consists of model-based approaches like Generalized Linear Models (GLM, see [80–83]) and others such as Linear-Nonlinear Poisson (LNP) cascade models [84–88] that explicitly model the relationship between the stimulus features and the neuron’s firing rate. The popularity of these models is based on their simplicity, on the robust convergence of parameter fitting, and on providing an explicit way to test how good our hypotheses about the neuron’s stimulus selectivity are.

Tensor-based methods like the ones explored here offer a complementary set of advantages to explicit models of stimulus selectivity. Tensor-based approaches model the statistics of the whole spike trains and try to identify directly (using linear assumptions) the response units within the firing patterns of a population which may then form the basis of different encoding or decoding models. However, they do not assume any relationship between the spike train and the stimuli. The activation coefficients of a tensor decomposition can have any arbitrary dependence, linear or non-linear, on any aspect of the physical stimulus presented to the animal. This property of tensor methods makes them potentially useful in cases in which there is no well-founded model or even clear hypothesis about the stimulus aspects that are encoded by the population. In the present study, this allowed us to analyze the spatiotemporal structure of retinal information under natural stimulation, for which no generally accepted, fully satisfactory model exists to date. Standard models such as GLMs and linear-nonlinear models that treat the receptive field as a single stimulus filter, do not capture the often nonlinear spatial stimulus integration by retinal ganglion cells [89–92] and therefore do not generalize easily to natural stimuli [93]. Moreover, these models do not capture a large proportion of variance for several classes of cortical cells when considering responses to natural stimuli [94–97]. Thus, although it was convenient to begin exploring and validating this methodology on retinal cells due to their response reliability and relative ease of recording of larger populations, it is important to continue validating this methodology on cortical responses in future studies, as this tool may become particularly valuable for cortical datasets not well described by standard stimulus-response models.

Importantly, both space-by-time NMF and orthogonal Tucker-2 had very strong generalization properties, as the space and time modules obtained from population responses to a set of stimuli could be used to describe the information carried by neural responses to other stimuli not used to train the algorithm. In addition to this decoding generalization advantage, space-by-time NMF was particularly competitive at retrieving non-orthogonal and overlapping firing patterns. The non-negativity constraints of space-by-time NMF have the evident advantage that the extracted patterns are directly interpretable as firing patterns and generalize well to patterns obtained for unseen stimuli. An emerging view on sensory population coding is that it is based on a limited range of temporally precise patterns of firing that are relatively stereotyped in shape and tend to partly overlap across stimuli [18–21]. The fact that space-by-time NMF copes well with these constraints underscores the utility and biological plausibility of space-by-time NMF as a tool to study sensory population codes.

### Efficiently decoding information and interpreting spatial and temporal modules as dictionaries of firing patterns forming population codes

Here we compared tensor and other decompositions for their ability in two partly competing requirements: the need to retrieve and describe the firing patterns of real spike trains robustly and reliably, and the ability to describe as much information as possible from spike trains with a compact basis function.

While we found that both the space-by-time NMF and orthogonal Tucker-2 perform well at both tasks (because of the advantages of the tensor factorization), we found that they excelled in different ways.

The advantage of the non-negative decomposition was its ability to robustly find compact and directly interpretable firing patterns that occur across many different kinds of stimuli. These patterns can be used as basis functions to linearly build a set of code-words of firing patterns, complementing existing approaches [98, 99]. The shape of these firing patterns can thus be examined to provide important information about the structure of the neural code, for example to make hypotheses about the spatial and temporal resolution at which a neural code should be read out. As an example of the information that these modules may give, the structure of spatial modules extracted from RGCs suggests that informative patterns of simultaneous firing come from localized groups of neurons whose receptive fields are close together and that have similar stimulus tuning. The structure of the temporal modules of RGCs when responding to static images highlighted the importance of measuring response latencies on a fine (10 ms) temporal scale (Figs 6A and 10C). The temporal modules in response to natural movies broke down movie scenes into temporal blocks, suggesting that RGC firing rates on a scale of (few) tens of milliseconds encode the dynamics of visual feature changes over the movie time course (Fig 7A). Moreover, the ability of space-by-time NMF to find the stereotypical firing patterns across stimuli led to a very good and robust decoding performance with a small set of basis functions, thus leading to a compact informative representation.

The main advantage of orthogonal Tucker-2 was the extremely high decoding performance it offered, albeit at the cost of a larger number of modules as compared to the more compact and sparser representations offered by space-by-time NMF. Its decoding advantage stems from the ability of orthogonal Tucker-2 to pick stimulus variations of firing patterns rather than their invariant properties. Its ability to decode information very efficiently, even when training the modules on responses to a different subset of stimuli and in the face of variations of module shapes across responses to different stimuli, can be expected in cases in which the variations of responses across sets of stimuli span a relatively low dimensional space and can thus be well captured by the orthogonal basis functions of orthogonal Tucker-2.

Which tensor decompositions should be used depends on the relative importance for the problem at hand. When the emphasis is purely on extracting sensory information, orthogonal constraints have a strong advantage. Non-negative constraints appear advantageous when the emphasis is both in obtaining good sensory information and also in identifying robust and functionally interpretable firing patterns. This is the case for example in experiments aimed at forming ideas about which neural population response patterns are important for information coding using statistical analyses of optically acquired neural population responses, and then testing these hypotheses about the information-bearing neural response patterns with optical stimulation of neural tissue [100].

### Role of spike timing in population coding

The ability of space-by-time decompositions to describe both the space and time dimension of neural responses makes this method useful for investigating how information in population codes distributes along these two dimensions. Taking advantage of this property, we developed a decoding approach that evaluates the relative contribution of space and time to the neural code by selectively destroying or preserving information along each dimension.

Application of this formalism to tens of RGCs in response to flashed images (sets of natural images differing by either fine or coarse spatial information, and gratings differing by spatial phase) and natural movies showed that, in general, both the spatial (i.e. differences across neurons of neural responses to stimuli) and the temporal structure of neural responses are key components of the neural code because they carry information that in general cannot be found in the other dimension. In the following we will discuss the implications regarding the time dimension of population codes, as there is considerable discussion regarding the role of temporal codes in representing sensory information [8, 9, 12, 15, 17, 101].

When analyzing responses to flashed images (natural images and gratings differing by spatial phase), we found that, while differences in image features on coarser spatial scales could be discriminated based on time-averaged responses, for both artificial and natural images, the neural population information about image details on a finer spatial scale could only be fully recovered from the precise spike times of RGCs on a 10-ms scale, but not from time-averaged responses. Given that the flashed images were held fixed for the stimulus presentation time, this represents evidence for temporal encoding [15, 102], that is, for the conversion of non-temporal (spatial) information into spike trains with distinct temporal structures. This finding is consistent with an influential theory that spike timing information of RGCs reflects local differences in stimulus intensities [68, 103] and suggests that this could be a primary source of temporal encoding of visual information, complementing other sources such as fixational eye movements [104].

Notably, first-spike latencies turned out to be a key part of spike timing information. Although total spike counts were sufficient to decode coarse image information from population activity, this coarse image information was also almost entirely available through first-spike latencies. Moreover, latencies carried more information than total spike counts about fine image features, always a large proportion of the total information contained in population spike trains, and were decodable without needing an external stimulus time reference. These results corroborate the idea that latencies form a dominant part of the retinal neural code for visual images allowing for rapid decoding of large amounts of visual information from population activity [12, 68, 103].

First-spike latencies had been shown to be a key component of the retinal code for fine details of artificial stimuli in small populations of few cells [12, 105]. Our results extended this previous work in two significant ways. First, we showed that spike timing and latencies are important also for coding fine spatial information in natural images. Second, the demonstration that latencies can be read out for larger populations of tens of cells shows that fluctuations of latencies are sufficiently coordinated across tens of cells to underlie robust image coding (something that was previously only shown to hold for cell pairs in [12]), and that the information carried by latency of one neuron is not redundant with the information carried by total spike count of another neuron. These results, which are important for establishing a key role of latencies in population activity, could be achieved because of the data robustness and the effectiveness of space-by-time NMF to capture information from limited datasets. This property was key to accurately compare first-spike latency information with the total information carried by this population both along the space and time dimensions.

Analysis of responses of RGCs to natural movies showed that spike timing carries information not available in total spike count also in this case, with the shape of temporal modules suggesting that spike timing carried information about the dynamics of visual features. All in all, our results suggest that in the retina, spike timing carries information both about image features and about their time evolution.

## Materials and Methods

### Ethics statement

All experimental procedures were performed in accordance with national and institutional guidelines and approved by the institutional animal care committee of the University Medical Center Göttingen.

### Decomposition methods

In the presented framework, we assume that data are composed of trials of spiking activity recorded simultaneously from a population of neurons in response to external stimuli. The two dimensions of these data are space (which neuron spiked) and time (when after stimulus onset did the neuron spike). Our goal is to find appropriate invariant modules, which are spatial, temporal or spatiotemporal patterns that capture the inherent structure of the responses, and trial-dependent coefficients that represent activation levels of those modules.

In the following, we describe methods for decomposing neural activity into such modules and activation coefficients. Depending on the assumptions made about the modular structure, different algorithms can be used to find the modules. Principal component analysis (PCA) assumes orthogonality of the modules, independent component analysis (ICA) assumes statistical independence of the modules, factor analysis (FA) assumes a specific latent variables model, and non-negative matrix factorization (NMF) assumes non-negativity of both modules and activation coefficients. Moreover, we adapt tensor decomposition methods with various constraints: orthogonal Tucker-2 applies orthogonality constraints, space-by-time NMF applies non-negativity constraints and Bayes Poisson Factor assumes negative binomial spike count distributions. All of these decompositions obtain modules that are constant across trials, with linear coefficients describing trial-to-trial activity, making them efficient to calculate and applicable to large populations.

Before applying any of the decomposition methods, we discretize neural responses by binning neural spike trains into short intervals (here 10 ms) and counting the number of spikes in each interval. We denote the neural spike counts on a single trial as **R**^*s*^, *s* ∈ {1, …, *S*}, where *S* is the total number of trials. For all *s*, **r**^*s*^(*t*) is the vector of population spike counts in bin *t*. Therefore, **R**^*s*^ ∈ ℝ^*T*×*N*^, *T* the number of bins per trial and *N* being the number of recorded neurons. *T* is constant across all trials, because here each trial has the same length.

#### Spatiotemporal decomposition models

In the so-called spatiotemporal decomposition [75], modules are full spatiotemporal neural activity patterns (similar to firing packets, [23]). In this model, neural activity **r**^*s*^(*t*) is decomposed into spatiotemporal modules as follows:

$$r^{s}(t)=\sum_{k=1}^{K}h_{k}^{s}b_{k}(t)\;+\text{ residual},$$ (1)

where *K* is the number of spatiotemporal modules **b**_*k*_ and $h_{k}^{s}$ is the activation coefficient of module *k*. Note that the modules **b**_*k*_ are trial-independent, whereas the activation coefficients $h_{k}^{s}$ do depend on the trial *s*. Each of the *K* modules is in ℝ^*T*×*N*^, yielding a total of *KTN* trial-independent parameters.

To calculate this decomposition, we represent the **b**_*k*_ as vectors **v**_*k*_ ∈ ℝ^1×*TN*^. Concatenating all *K* vectors yields a matrix **B** ∈ ℝ^*k*×*TN*^. Consequently we represent all **R**^*s*^ as a matrix **R** ∈ ℝ^*S*×*TN*^ and all $h_{k}^{s}$ as a matrix **H** ∈ ℝ^*S*×*K*^. Now we need to decompose **R** into **B** and **H**:

R=HB + residual. (2)

We can apply standard dimensionality reduction techniques to obtain linear decompositions of this equation and afterwards reshape **B** and **H** to obtain all **b**_*k*_ and $h_{k}^{s}$. We consider four different decomposition algorithms for solving Eq (2): PCA, ICA, FA and NMF.

PCA chooses the first module to maximize the variance of all observations along the module axis. The remaining modules are then selected subsequently. The *n*-th module axis is orthogonal to the previous *n*-1 module axes and chosen to maximize the variance of all observations among all possible choices of the *n*-th module axis [106]. We applied MATLAB’s *princomp* function to obtain PCA modules and activation coefficients (MATLAB and Statistics Toolbox Release 2014a, The MathWorks, Inc., Natick, Massachusetts, United States).

ICA estimates modules that are non-Gaussian and mutually independent [107]. We used Hyvärinen's fixed-point iteration scheme to find these modules and associated activation coefficients [108].

FA applies a statistical model with latent variables, called common factors, and observed variables modelled as linear combinations of the hidden common factors. In addition, each observed variable is assumed to be distorted by an independent Gaussian noise term [56–58]. We used Ghahramani's formulation of factor analysis which applies the expectation maximization algorithm to learn all parameters of the FA model [57] and thereby obtained a factorization in the form of Eq (2).

NMF decomposes a matrix **R** into non-negative modules captured in **B** and non-negative activation coefficients captured in **H**. For neural activity, non-negativity is a natural constraint since spike counts are non-negative by definition. Moreover, NMF-based methods yield parts-based and sparse representations [49]. We applied the multiplicative update rule for minimizing the Euclidian norm to obtain the NMF decomposition [109].

#### Space-only decomposition models

In order to obtain spatial PCA, ICA, FA and NMF modules, we performed a space-only decomposition. In this decomposition, the neural activity **r**^*s*^(*t*) is decomposed into trial-independent spatial modules and trial-dependent activation coefficients as follows:

$$r^{s}(t)=\sum_{k=1}^{K}h_{k}^{s}(t)b_{k}\;+\text{ residual},$$ (3)

where *K* is the number of spatial modules **b**_*k*_ and $h_{k}^{s}$ is the time- and trial-dependent activation coefficient of module *k*., where now, each spatial module **b**_*k*_ is an *N*-dimensional vector. To calculate this decomposition, we concatenate the **b**_*k*_ to a matrix **B** ∈ ℝ^*K*×*N*^ and represent all **R**^*s*^ as a matrix **R** ∈ ℝ^*ST*×*N*^ and all $h_{k}^{s}(t)$ as a matrix **H** ∈ ℝ^*ST*×*K*^. This yields again Eq (2), which we solve using PCA, ICA, FA or NMF.

#### Space-by-time decomposition models

The core underlying assumption of the space-by-time decomposition model is that all spatiotemporal modules can be factorized into spatial and temporal modules. It extracts spatial and temporal modules separately but concurrently. In this model, the neural activity **r**^*s*^(*t*) is decomposed into components as follows:

$$r^{s}(t)=\sum_{i=1}^{P}\sum_{j=1}^{L}b_{i}^{\text{tem}}(t)h_{i,j}^{s}b_{j}^{\text{spa}}\;+\text{ residual},$$ (4)

where *P* is the number of temporal modules *b*^tem^, *L* is the number of spatial modules $b_{j}^{\text{spa}}$, and $h_{i,j}^{s}$ is the activation coefficient of modules *i* and *j* in trial *s*. Note that, again, temporal and spatial modules are trial-independent, whereas the activation coefficients are trial-specific.

Eq (4) can be written in matrix notation as a 3-matrix factorization:

$$R^{s}=B^{\text{tem}}H^{\text{s}}B^{\text{spa}}\;+\text{ residual},$$ (5)

where **B**^tem^ ∈ ℝ^*T*×*P*^ is a matrix whose columns are the temporal modules, **B**^spa^ ∈ ℝ^*L*×*N*^ is a matrix whose rows are the spatial modules and the matrix $H^{s}={\left(h_{i,j}^{s}\right)}_{\begin{array}{l} 1\leq i\leq P\\ 1\leq j\leq L \end{array}}$ includes all single-trial activation coefficients.

Yet another way to write Eq (4) is the following tensor factorization:

$$R=Η\times_{1}{\left(B^{\text{tem}}\right)}^{\text{T}}\times_{2}B^{\text{spa}}\;+\text{ residual},$$ (6)

where in this equations **R** denotes the *T* × *N* × *S* tensor composed of all **R**^*s*^, **H** denotes the *P* × *L* × *S* tensor (called core tensor) composed of all **H**^*s*^, ×_*n*_ denotes the *n*-mode tensor-matrix product and (**B**^tem^)^T^ denotes the transpose of **B**^tem^. This tensor decomposition is known as the Tucker-2 tensor factorization [50, 51].

For the Tucker-2 tensor factorization with non-negative constraints (i.e. space-by-time NMF), we applied iterative update rules that were derived independently under the name “Non-negative Tucker-2 decomposition” in [60] and in the context of muscle synergies under the name “sample-based non-negative matrix tri-factorization” (sNM3F) in [59]. Here, we present the update rules as described in [59]. These rules iteratively minimize the Frobenius norm of the difference between the input data and the reconstructed data $\sum_{s=1}^{S}{\left\|R^{s}-B^{\text{tem}}H^{s}B^{\text{spa}}\right\|}^{2}$. The updates rules cannot increase the reconstruction error [59]. Therefore, the algorithm is guaranteed to converge to a local minimum. The optimization problem is not convex, so the local minimum is not necessarily global. This also implies that the modules found are not unique. Empirically, however, the modules tend to be very similar from one algorithm run to the next (S9 Fig). Previously, this was also noted by other studies using NMF [110].

The complete space-by-time NMF algorithm takes the following form [59]:

1. Initialize **B**^tem^(*T* × *P*), **H**(*P* × *L* × *S*), and **B**^spa^(*L* × *N*) with positive random numbers uniformly distributed between 0 and 1.
2. Given **H**, **B**^tem^ and the data matrix **R**(*N* × *T* × *S*), update **B**^spa^:
  - Reshape **H**→**H**(*P* × *LS*)
  - Calculate **G** = **B**^tem^**H**
  - Reshape **G**→**G**(*TS* × *L*) and **R**→**R**(*TS* × *N*)
  - For all *i* ∈ {1, …, *L*}, *j* ∈ {1, …, *N*}:
    $$B_{i,j}^{\text{spa}}\leftarrow B_{i,j}^{\text{spa}}{\left(G^{\text{T}}R\right)}_{i,j}/{\left(G^{\text{T}}GB^{\text{spa}}\right)}_{i,j}$$
3. Given **H**, **B**^spa^ and **R**, update **B**^tem^:
  - Reshape **H**→**H**(*PS* × *L*)
  - Calculate **V** = **HB**^spa^
  - Reshape **V**→**V**(*P* × *NS*) and **R**→**R**(*T* × *NS*)
  - For all *i* ∈ {1, …, *T*}, *j* ∈ {1, …, *P*}:
    $$B_{i,j}^{\text{tem}}\leftarrow B_{i,j}^{\text{tem}}{\left(RV^{\text{T}}\right)}_{i,j}/{\left(B^{\text{tem}}VV^{\text{T}}\right)}_{i,j}$$
4. Given **B**^tem^ and **B**^spa^ and **R**, update **H**:
  - For all *s* ∈ {1, …, *S*}:
    Define **H**^*s*^(*P* × *L*) and **R**^*s*^(*N* × *T*)
  - For all *i* ∈ {1, …, *P*}, *j* ∈ {1, …, *L*}:
    $$H_{i,j}^{s}\leftarrow H_{i,j}^{s}{\left({\left(B^{\text{tem}}\right)}^{\text{T}}R^{s}{\left(B^{\text{spa}}\right)}^{\text{T}}\right)}_{i,j}/{\left({\left(B^{\text{tem}}\right)}^{\text{T}}B^{\text{tem}}H^{s}B^{\text{spa}}{\left(B^{\text{spa}}\right)}^{\text{T}}\right)}_{i,j}$$
5. If decrease in $\sum_{s=1}^{S}{\left\|R^{s}-B^{\text{tem}}H^{s}B^{\text{spa}}\right\|}^{2}$ is below a given tolerance, normalize **B**^tem^ row-wise, **B**^spa^ column-wise, rescale **H** to make the error unchanged and stop. Otherwise, go to step 2.

The Tucker-2 tensor factorization with orthogonal constraints can be implemented by iteratively computing left singular vectors [50, 51]. Here, we applied the *tucker* function from the N-way toolbox for MATLAB [111] to compute this tensor decomposition.

Bayes Poisson Factor [63] yields a decomposition like that provided by the Tucker-2 but with a) the same number of modules for each factor and b) no interactions between factors, i.e. the core tensor is restricted to be the identity tensor. Bayes Poisson Factor shares these properties with PARAFAC which is a special case of Tucker decompositions. However, Bayes Poisson Factor also applies non-negativity constraints by means of a stochastic model involving negative binomial distributions which have non-negative support. Here, we applied the Bayes Poisson Factor toolbox for MATLAB [63] to compute this tensor decomposition.

Fig 1 illustrates the difference between the spatiotemporal methods and the space-by-time methods. We show the decomposition of two trials (blocks in cyan). The spatiotemporal methods decompose the trials into spatiotemporal modules and corresponding activation coefficients. On the other hand, the space-by-time methods decompose the trials into products of space and time modules. In both cases, linear mixtures of modules reconstruct the original trials. In the figure, the resulting product modules illustrate the similarity of the effective modules to the spatiotemporal modules.

MATLAB code for these methods is also available at http://stommac.eu/index.php/code.

### Multielectrode recordings from retinal ganglion cells

We extracellularly recorded spike trains from retinal ganglion cells in the isolated salamander retina with multielectrode arrays [112] as described previously [113]. In brief, retinas were isolated from dark-adapted *axolotl salamanders* (Ambystoma mexicanum; pigmented wild type) of either sex and placed ganglion-cell-side-down on a 252-channel multielectrode array (Multichannel Systems, electrodes of 10 μm diameter with a spacing of 60 μm). All experimental procedures were performed in accordance with institutional guidelines of the University Medical Center Göttingen. Recordings were performed at room temperature while supplying the retina with oxygenated Ringer’s solution. Spikes were detected and sorted by an expectation-maximization algorithm for a Gaussian mixture model [114]. For each recorded cell, receptive fields were determined by computing the spike-triggered average from stimulation with spatiotemporal white noise [85]. Singular value decomposition was used to separate the spike-triggered average into a spatial and temporal component [115]. Finally, a two-dimensional Gaussian function was fitted to the spatial receptive field component to determine the center, size, and shape of the receptive field.

Visual stimuli were projected onto the photoreceptor layer of the retina by a gamma-corrected miniature OLED display (600 × 800 pixels) with monochromatic white light. A telecentric lens demagnified the stimuli to a pixel size of 7.5 μm × 7.5 μm. Average light intensity for all stimuli on the retinal surface was approximately 2.6 mW/m^2^, in the photopic range, on the retinal surface. Stimulus presentation was controlled through a custom-made software package, based on C++ and OpenGL. For the purpose of analyzing population spike patterns, we applied three types of stimuli whose details are given below: flashed natural images, natural movies, as well as flashed gratings. Data were analyzed from a total of five retinas, comprising 38, 49, 54, 23 and 37 ganglion cells, respectively. Natural images and movies were presented in experiments 1 and 2; gratings were presented in experiment 3. Natural images with shifts were presented in experiments 4 and 5.

### Stimulation with flashed natural images and gratings

We selected a set of 60 natural photographs from the “McGill Calibrated Colour Image Database” http://tabby.vision.mcgill.ca/html/browsedownload.html [116]. The images are shown as an overview in S11 Fig. They display a wide range of natural and artificial scenes, all consistent with spanning a field of view of around 20° to 40°. Each image has a spatial resolution of 256 × 256 pixels, covering a total area of 1920 μm × 1920 μm on the retina. The provided RGB-color values for each image were converted into grayscale by applying a weighted average over the three color channels and normalizing by the known exposure time of the camera, so that the resulting pixel values are linearly related to the absolute luminance values of the original input image. Mean and standard deviation of the pixel values were normalized for each image by appropriately shifting and scaling the values so that the standard deviation was set to 50% of the mean intensity. Pixel values that then deviated from the mean by more than 100% in either direction were clipped to ensure that the maximal pixel values are within the physically available range of the display. To minimize the artifacts induced by this clipping, we only selected images that had only few clipped pixels (i.e., not more than 0.035% of the pixels). Images were presented individually for 200 ms each in a pseudo-random sequence, with an inter-stimulus-interval of 800 ms in which a full field gray stimulus was presented. For data analysis, we used the first 300 ms of neural activity after stimulus onset. We recorded data from two retinas for this experiment (n = 38 and n = 49 cells, respectively). At least 30 trials per image were recorded.

In a second set of experiments (flashed gratings with different spatial phases), we used square-wave gratings of 900 μm spatial period (thus a bar width of 450 μm, a little larger than the typical size of a RGC RF) and 60% visual contrast. The stimulus set consisted of 60 shifted versions of the same grating, uniformly covering the complete range of spatial phases of the grating. The step of spatial shifts from one image to the next was thus much smaller than the typical RF size of RGCs. Stimulus presentation and data analysis proceeded analogous to the case of natural images. We recorded data from one retina for this experiment (n = 54 cells). At least 30 trials per grating were recorded.

In a third set of experiments (different natural images presented with different small shifts), we selected a subset of 9 natural photographs from the “McGill Calibrated Colour Image Database” [116], with photographs converted from RGB to grayscale as explained above. The stimulus set consisted of the presentation of these images shifted in 9 different possible positions. The shifts were arranged so that the image center points lie on an orderly 3-by-3 lattice (as illustrated in Fig 11, where we show different shifts used for one experiment), with 90 μm between neighbors. The 90 μm step of these shifts was much smaller than the size of a typical RGC RF. Also in this case, images were presented individually for 200 ms each in a pseudo-random sequence, with an inter-stimulus-interval of 800 ms, during which a full field gray stimulus was presented. Each different shifted image was presented 19 times. For data analysis, we used the first 300 ms of neural activity after stimulus onset. We recorded data from two retinas for this experiment (n = 23 and n = 37 cells, respectively).

### Stimulation with natural movies

Two natural movies were selected for presentation to the retina. The first movie (“salamander movie”) contains scenes of salamanders swimming in a tank. The second movie (“tiger movie”), containing wild life scenes of a tiger on a pray hunt, was produced by BBC Documentary. Both movies were obtained from YouTube.com. The two movies were roughly 60 s in duration and were repeated at least 30 times. Example still frames from both movies are shown in S5 Fig. Similarly to the natural images, the movies were converted to grayscale, with a spatial resolution of 360 × 360 pixels at 7.5 μm × 7.5 μm per pixel, covering a total area of 2700 μm × 2700 μm on the retina. Mean light intensity and contrast were normalized to be the same as for the natural images.

For analyzing the data obtained with natural movies, we partitioned each movie and corresponding neural activity into 60 scenes of length 300 ms and used only the first 30 trials for analysis (the same length and number of trials that we used for analyzing image responses). In order to construct a mixed set of scenes that were challenging both in terms of timing and in terms of spatial information, each set of movie scenes was composed of two subsets. The first subset of non-overlapping scenes was selected such that, based on their total spike counts, these scenes were indistinguishable and each scene contained at least 3 cells that elicited at least one spike in all trials. We found between 6 and 16 scenes per experiment that fulfilled these criteria. To complete the total number of 60 scenes per movie, remaining scenes in the second subset were selected randomly such that all resulting scenes were non-overlapping. We recorded data from two retinas for this experiment (n = 38 and n = 49 cells, respectively, the same ones that we recorded in response to the natural images dataset).

### Decoding analysis

We used multiclass linear discriminant analysis (LDA) [117] in conjunction with a training set—test set procedure to predict the presented stimulus identity (images or movie scenes, identity between 1 and 60) in each trial using the single-trial activation coefficients of each model. For each experiment with natural images, natural movies and gratings, we analyzed a total of 30 data trials per stimulus. We randomly separated these trials into an equal number of 15 training trials and 15 test trials. For each of the two experiments with shifted natural images, we analyzed a total of 19 data trials per stimulus. We randomly separated these trials into 10 training trials and 9 test trials. The training trials for all stimuli formed the training set and the test trials for all stimuli formed the test set. For each algorithm (spatiotemporal PCA, ICA, FA, NMF, orthogonal Tucker-2, space-by-time NMF and Bayes Poisson Factor), we calculated modules and training activation coefficients on the training set. For these given modules, we then calculated the activation coefficients on the test set. We removed activation coefficients with zero variance across all trials from the analysis. We then trained the LDA classifier on the activation coefficients of all training trials and evaluated performance of the LDA classifier on the test trials to estimate the generalization error [118].

There are many options for selecting the numbers of spatial and temporal modules. Delis et al. selected these numbers by greedily increasing the number of spatial and temporal modules until decoding performance did not increase significantly any further [59, 119]. Various other stopping criteria for adding modules have also been proposed based on a measure called “Variance Accounted For” [119] including fixed thresholds [120], the point at which the highest change in slope is observed [73] and the point at which the curve plateaus to a straight line [119, 121].

At heart, selecting the numbers of modules is a model selection problem which can also be solved with general cross-validation methods [118, 122–124]. In this study, we followed the common leave-one-out cross-validation method for its conceptual simplicity, computational inexpensiveness and good performance compared to other model selection techniques [124]. We did this by varying the numbers of spatial and temporal modules. We emphasize that we used only the training set for selecting the optimal number of modules: One trial (of each stimulus) of the training set is used as the validation set and the remaining trials of the training set are used to train the modules. This is repeated for each trial, yielding a validation performance for each training trial and each number of spatial and temporal modules. We then average over trials to obtain an average validation performance for each number of spatial and temporal modules (S4C Fig). We then selected those numbers with the maximum average decoding performance on the validation set (c.f. Fig 4C, S4C Fig). If more than one pair of numbers attained the maximum performance then we selected the pair with maximum performance and minimum sum of module numbers. Ultimately, the optimal number of parameters turned out to be very robust and easy to determine.

For comparison, we also evaluated the performance of LDA when applied to the discretized neural responses without any dimensionality reduction (here referred to as “raw LDA”). For this purpose, we represented the *N* × *T*-dimensional spike count matrix of each trial as a *NT* × 1-dimensional vector and used all entries of this vector as predictors for the LDA classifier. We then used the same hold-out cross-validation procedure as in the case of the other algorithms. We quantified decoding performance as the percentage of correct predictions on the test set.

## Supporting Information

S1 Text Additional information regarding the analysis procedures. (PDF)

S1 Fig Recovering firing patterns from simulated data by spatiotemporal ICA, spatiotemporal FA and Bayes Poisson Factor. Figure conventions as in Fig 2. (A) A case when the ground truth modules can be factorized into space and time. Top row: Four ground truth modules for generating spike trains. Inhomogeneous Poisson spike trains are generated with a background rate (white) and a stronger foreground rate (red). The red blocks fire with high SNR (300 Hz vs. a background rate of 2 Hz). Each row shows the modules that were recovered by the denoted method. (B) As in panel A but with ground truth patterns made of blocks with lower SNR (30 Hz vs. background rate of 2 Hz). (C) A case of decomposition of high firing rate patterns that are not separable in space and time.
(TIF)

S2 Fig Generation of artificial data and illustration of artificial stimulus conditions. (A) Inhomogeneous Poisson spike trains are generated with a background rate (white) and a stronger foreground rate (red). The foreground blocks appear randomly and (possibly) together. In the shown example, the overlap is 32.25%. Middle panels show example raster plots. Each raster plot represents one trial. Bottom panels show discretization of the corresponding spike raster plots. The number of spikes in each bin is counted to produce the gray value blocks. (B) Patterns of overlapping blocks are combined to create 6 different stimulus conditions. Blocks from overlap 3 (Fig 4A) are shown in the left column. In each stimulus condition (right column), exactly two blocks are present. The selection of blocks is constant for a given stimulus and therefore characterizes the stimulus condition.
(TIF)

S3 Fig Orthogonal Tucker-2 based stimulus decoding of simulated data. (A) Stimulus decoding performance on simulated data constructed like in Fig 4A and 4B with varying number of trials per stimulus and signal-to-noise ratio (SNR) obtained using orthogonal Tucker-2. (B) Percentage of correct selection of the number of modules as a function of the number of trials per stimulus for orthogonal Tucker-2. We selected the smallest numbers of modules with the maximum test set decoding performance and compared the selected numbers to the ground truth numbers (2 temporal and 2 spatial modules).
(TIF)

S4 Fig Selection of the optimal number of spatial and temporal modules. (A) Average leave-one-out validation set decoding performance with SNR = 20 and number of trials (training+test) per stimulus = 30 for spatiotemporal NMF (top row) and for space-by-time NMF (bottom row) is shown. Overlap of the patterns is increasing from left to right (overlap 0 to 3). We select the smallest numbers of modules with the maximum validation decoding performance (white squares—also corresponding to the ground truth: 4 spatiotemporal modules, 2 temporal and 2 spatial modules). (B) Average decoding performance for an example experimental session on the training set averaged over leave-one-out cross-validation sub-samples for different numbers of temporal modules (x-axis) and spatial modules (y-axis). (C) Like B but for the validation set. The smallest numbers of modules with the maximum average validation set performance are selected (white square: 8 spatial modules, 3 temporal modules).
(TIF)

S5 Fig Movie autocorrelations. (Top left) Trial still frames from both movies. (Top right) Autocorrelation of the salamander movie (dashed blue) and tiger movie (dashed black) and corresponding salamander and tiger movie autocorrelation fits (solid blue and black, respectively). (Bottom) Equations of the autocorrelation fits.
(TIF)

S6 Fig Eight spatial modules that were identified by decomposition methods. The modules are represented as receptive fields. Cyan represents positive module amplitude and magenta represents negative module amplitude. The more saturated the color the stronger the absolute amplitude of the neuron in the module. (A) Modules identified by PCA. (B) Modules identified by ICA. (C) Modules identified by FA. (D) Modules identified by NMF.
(TIF)

S7 Fig Spatial and temporal modules that were identified by orthogonal Tucker-2. Representation of the modules as in S6 Fig and for the same image dataset. (A) Modules that were identified by orthogonal Tucker-2 for numbers of modules that were optimal for space-by-time NMF to facilitate comparisons with Fig 6 and S6 Fig. (B) Modules that were identified by orthogonal Tucker-2 for numbers of modules that were optimal for orthogonal Tucker-2. The optimal numbers of modules are greater than for space-by-time NMF.
(TIF)

S8 Fig Module recovery similarity of modules recovered by orthogonal Tucker-2 and space-by-time NMF. Geodesic similarity between the modules recovered for the full number of trials per stimulus and the modules recovered for a lower number of trials for orthogonal Tucker-2 (magenta) and space-by-time NMF (blue) as a function of the number of trials per stimulus averaged over all image datasets (A, B) or all movie datasets (C, D) for temporal modules (A, C) or spatial modules (B, D).
(TIF)

S9 Fig Stability of modules over stimulus sets for one representative session with image stimuli. (A) Temporal (top) and spatial (bottom) modules that were obtained by training on data in response to 1 (left), 5 (middle), and 10 (right) training images, drawn randomly from the complete set of 60 stimuli and repeated 10 times to calculate averages and standard deviations. (B) Examples of original trial recordings and reconstructions with different numbers of stimuli for training the space-by-time NMF modules. Neural activity is shown as gray-value bins. The darker the bin the more spikes are present in that bin. Stimuli for the respective row are shown on the left. “Recording” shows the original trials. 1, 5, and 10 stimuli reconstruction show the reconstructions based on space-by-time modules that were trained on 1, 5, and 10 stimuli respectively which do not include the shown stimuli.
(TIF)

S10 Fig Illustration of shuffling procedures and demonstration of relative importance of spike timing and single-neuron firing rates on simulated data. (A) Original spatiotemporal trial. Each block represents a bin. Each line represents a spike. A synthetic population response is shown for 5 neurons N1-5 and 9 time bins t1-9. (B) Space-only condition: responses are permuted across time bins t1-9. Thereby, timing information is destroyed. (C) Time-only condition: responses are permuted across neuron identity. Thereby, spatial information is destroyed. (D) Patterns for spike train generation are plotted with the same conventions as in Fig 4A. Pattern 1 has information in space and time. Pattern 2 has information in space only and pattern 3 has information in time only. The areas of the patterns are kept constant across all three conditions. (E) Stimulus decoding performance of space-by-time NMF on these simulated data with varying number of trials per stimulus and SNR after training on unshuffled responses (space-and-time, top row), after shuffling bins across time (space-only, center row) and after shuffling bins across neurons (time-only, bottom row). SNR as in Fig 4B.
(TIF)

S11 Fig Overview of the set of 60 photographs that were used as natural image stimuli. Images were selected from the “McGill Calibrated Colour Image Database” http://tabby.vision.mcgill.ca, converted into grayscale. Mean and standard deviation of the pixel values were normalized for each image.
(TIF)

S12 Fig Variation of image decoding difficulty to assess importance of time and space coding. Randomly sampled subsets of the total number of neurons (*N*) are used for decoding image identities. (A) Decoding performance as a function of the population size for unshuffled responses (space-and-time), responses shuffled across time (space-only) and responses shuffled across cells (time-only). (B) Relative decoding loss as a function of the population size of the respective shuffling method compared to the unshuffled condition.
(TIF)

S13 Fig Orthogonal Tucker-2 analysis of responses to flashed gratings and to shifted natural images. Comparison of decoding performance after training on unshuffled responses with orthogonal Tucker-2 (space-and-time), after shuffling bins across time (space-only), and after keeping only the first spike of each neuron in each trial (latency-code), again after training on responses with orthogonal Tucker-2. We also included the rank order performance from Fig 10D for comparison. (A) Performance on flashed gratings dataset. (B) Performance of decoding image id and image position. (C) Performance of decoding image id for each position, averaged over positions. (D) Performance of decoding image position for each image id, averaged over images. ***p<0.001; two-tailed t-test. Error bars indicate s.e.m.
(TIF)

S14 Fig Effective temporal precision of flashed gratings and shifted natural image datasets. Decoding performance obtained from responses sampled at different effective precisions for the flashed gratings dataset (A) and for the shifted natural image datasets (B-D). The effective precision of 10 ms corresponds to the performance for the unshuffled responses. The effective precisions of 20, 50, 100 and 300 ms were obtained by shuffling bins in 2, 5, 10 and 30 neighboring bins, respectively. (B) Performance of decoding image id and image position. (C) Performance of decoding image id for each position, averaged over positions. (D) Performance of decoding image position for each image id, averaged over images. *p<0.05; ***p<0.001; two-tailed t-test. Error bars indicate s.e.m.
(TIF)

S15 Fig Spiking activity within and across modules. (A) Top: Presented stimuli of the respective column. (Middle) The blue frame marks neurons that belong to the same module (neurons 7, 9, 10). Each row shows raster plots of a single neuron. (Bottom) Raster plots of three neurons belonging to different modules (neurons 5, 13, 15). (B) Noise correlations within and across modules averaged over all image and movie datasets. A pair of neurons is in the “within modules” group if their amplitudes within a module are above a threshold that is set to have half of the pairs in the “within modules” group. ***p<0.001; one-tailed t-test. Error bars indicate s.e.m. (C) As in B, but for signal correlations.
(TIF)

S16 Fig Space-by-time NMF activation coefficients for one natural images example session. Each panel shows the activation coefficients corresponding to one temporal-spatial module pair (i.e. one element in the **H**^*s*^ matrix) as a function of the trial index *s*. The dashed red lines separate natural image stimuli: activation coefficients between two dashed red lines belong to trials of the same natural image (15 trials per image in the training set). One can easily appreciate the selectivity of activation coefficients to particular natural images. Many coefficients are zero for particular natural images, indicating moderate sparseness of the coefficient matrices.
(TIF)

S17 Fig Comparison of the space-and-time information computed with spatiotemporal or space-by-time NMF in the flashed gratings and to shifted natural images. (A) Performance on flashed gratings dataset. (B) Performance of decoding image id and image position. (C) Performance of decoding image id for each position, averaged over positions. (D) Performance of decoding image position for each image id, averaged over images. ***p<0.001; two-tailed t-test. Error bars indicate s.e.m.
(TIF)
