# Supplementary material for: Using Matrix and Tensor Factorizations for the Single-Trial Analysis of Population Spike Trains
Source: PLoS Comput Biol. 2016 Nov 4;12(11):e1005189. doi: 10.1371/journal.pcbi.1005189 (PMC5096699; doi:10.1371/journal.pcbi.1005189)
Supplement: S1 Text — (PDF) [file pcbi.1005189.s001.pdf]

Supporting Information S1 Text

Simulated spike train datasets

To test and compare the methods on data for which we know the ground truth, we generated simulated data by drawing Poisson spike trains with spatiotemporal patterns given by underlying firing rates and then binning the spike trains. This data generation process is illustrated in S2A Fig.

We used four spatiotemporal patterns (blocks or stripes), expressed as low ("background", white region in S2A Fig) and high ("foreground", red region in S2A Fig) firing rates of subsets of neurons at given fixed times within the trial. We used a background firing rate of 2 Hz on all trials and spatiotemporal pattern foreground rates of 30 Hz and 300 Hz per pattern. For simulations presented in Fig 4, we also varied the foreground rate as a function of signal-to-noise ratio (foreground rate = SNR × background rate). The spatiotemporal patterns that we used had overlapping regions in order to introduce statistical dependencies between the patterns. On each trial, each of the four spatiotemporal patterns had a 50% probability of being present on that trial. When more than one pattern was present on a given trial then the foreground rates of all present patterns on that trial were summed together resulting in higher effective foreground firing rates in the overlapping regions. Each simulated trial consisted of spike trains for 10 neurons and 200 ms.

We actually generated these spike trains in the following way: We generated the Poisson trains independently of each other by first drawing the number of spikes in each time bin from a Poisson count distribution with an appropriate rate and then positioning the spike times with a uniform probability distribution over the respective interval.

Quantification of similarity between modules

On both simulated and real spike train datasets, we quantified the similarity between modules using geodesic similarity instead of Euclidean similarity. Contrary to Euclidean similarity, the geodesic similarity better reflects the geometry of low-dimensional manifolds that are embedded in a high-dimensional space [1] which is important in the context of dimensionality reduction.

Formally, we define similarity between a pair of spatiotemporal modules as the geodesic similarity between the vectorized modules $\mathbf{b}_1$ and $\mathbf{b}_2$:

$$o_2\left(\mathbf{b}_1,\mathbf{b}_2\right)=\left(1-\frac{2}{\pi}\cos^{-1}\left\langle \mathbf{b}_1,\mathbf{b}_2\right\rangle\right)\cdot 100\%, \qquad \text{(S1)}$$

where $\langle\cdot,\cdot\rangle$ denotes the inner product. For non-negative modules, the resulting similarity o_2 is a value between 0% and 100%. When the module entries can be negative, as in the orthogonal Tucker-2 decomposition, then o_2 in Eq. (S1) is a value between -100% and 100%. To adjust for this problem and obtain a non-negative geodesic similarity measure that allows a fairer comparison with geodesic similarity values calculated over non-negative modules, in such signed-module cases we calculated o_2 of Eq. (S1) by considering modules $\mathbf{b}_i$ and $-\mathbf{b}_i$ as being equivalent, and setting the sign of the modules so that their geodesic similarity is non-negative.

For evaluating module recovery similarity between a set of ground truth modules $\mathbf{b}_1,\ldots,\mathbf{b}_n$ and a set of recovered modules $\mathbf{c}_1,\ldots,\mathbf{c}_n$ we calculated:

$$o_{\text{recovery}}\left(\mathbf{b}_1,\ldots,\mathbf{b}_n;\mathbf{c}_1,\ldots,\mathbf{c}_n\right)=\frac{1}{n}\sum_{i=1}^{n}o_2\left(\mathbf{b}_i,\mathbf{c}_i\right) \quad \text{(S2)}$$

where we ordered the modules $\mathbf{c}_1,\ldots,\mathbf{c}_n$ using a greedy algorithm to maximize similarity to the set $\mathbf{b}_1,\ldots,\mathbf{b}_n$.

For evaluating the overlap within a single set of modules $\mathbf{b}_1,\ldots,\mathbf{b}_n$ we calculated the geodesic similarity averaged across all module pairs:

$$o_{\text{overlap}}\left(\mathbf{b}_1,\ldots,\mathbf{b}_n\right)=\frac{2}{n(n-1)}\sum_{i=2}^{n}\sum_{j=1}^{i-1}o_2\left(\mathbf{b}_i,\mathbf{b}_j\right) \quad \text{(S3)}$$

The calculation of overlap from simulated data was straightforward because we knew the ground truth modules that we inserted in the simulation. For the calculation of the overlap from experimental data, we relied instead on the modules empirically estimated from the data, as explained in the next section.

Measure of SNR and overlap of modules in real retinal data

In the experimental datasets, we calculated the SNR for each stimulus as the foreground rate, estimated over the 100-200 ms interval after stimulus onset, divided by the background rate, estimated in the interval from 0-50 ms after stimulus onset, and averaged over neurons and trials. We discarded stimuli without background spikes of any neuron in any trial in order to avoid infinite SNR. However, we did not discard neurons or trials without spikes in general. We found that most neurons did not fire at all in the 0-50 ms interval which is why we found high SNRs. For experiments 1 to 3, our procedure yielded average SNRs of 74 (standard-error: 12), 147 (standard-error: 29) and 621 (standard-error: 98), respectively, indicating that the SNR in the experimental data is more than sufficient for good decoding performance.

We could not directly evaluate overlap of modules in the experimental datasets, because the ground truth modules were unknown. In order to obtain an estimate of the overlap of modules, we used the modules that were recovered by space-by-time NMF. We quantified the overlap by means of the geodesic similarity averaged across all module pairs, as described in Section "Quantification of similarity between modules". Using this method, the retinal ganglion cell datasets yielded overlap values between 0.16% and 3.75%.

Permutation procedure to identify the relative importance of space and time

Information obtained with space-by-time NMF from population spike trains contains both the information carried by the spatial and the temporal dimension of neural activity. We quantified the contributions of these two dimensions to the total information using the following permutation procedure (c.f. S10 Fig).

First, we applied space-by-time NMF to the unmodified single trial population responses. Like before, we decoded the presented stimuli from the single-trial coefficients of the NMF decompositions by first computing the NMF modules and training an LDA stimulus classifier on a training set (half of the trials) and then using the classifier to decode the stimuli presented on a test set (remaining half of trials) of

activation coefficients (c.f. Materials and Methods: Decoding Analysis). This procedure recovers information from both space and time. Hence, we refer to the recovered information as “space-and-time information”.

We investigated the contribution of space to the space-and-time information by randomly permuting each single-trial population response along the time dimension without permuting responses along the space dimension (S10B Fig). After the permutation step, we decoded the presented stimuli from the single-trial coefficients of the NMF decompositions as before by first computing the NMF modules and training an LDA stimulus classifier with the same training set / test set procedure. Due to the time-permutation step, this procedure recovers information carried by the neuron-to-neuron difference in firing rates only while losing all information in the temporal dimension. Hence, we refer to the recovered information as “space-only information”.

Similarly, we investigated the contribution of the temporal dimension to the space-and-time information by randomly permuting each single-trial population response along the spatial dimension without permuting responses along the temporal dimension (S10C Fig). Again, after the permutation step, we decoded the presented stimuli from the single-trial coefficients of the NMF decompositions using the same training set / test set. Due to the space-permutation step, this procedure recovers information carried by the temporal structure only while losing all information in the spatial dimension. Hence, we refer to the recovered information as “time-only information”.

To test the effectiveness of this permutation procedure to quantify the contributions of space and time, we constructed simulated spike trains in response to different simulated “stimulus” conditions with different spatiotemporal response patterns. The evaluation procedure follows the one that we used for investigating decoding performance of space-by-time NMF for spatiotemporal activity patterns with different overlaps (c.f. Fig 4). Again, we used spatiotemporal patterns of strong activity of subgroups of neurons (“blocks”) to generate Poisson spike trains and simulated responses to 6 stimulus conditions, with responses in each condition made up of a combination of two different blocks (S2B Fig), but this time we constructed the activity patterns in such a way as to probe the contributions of space and time.

We considered three simulated different cases. In case 1 (S10D Fig left), the blocks differed in both the spatial and the temporal dimension. In this case therefore information was present in both space and time. In case 2 (S10D Fig center) the blocks differed in the spatial dimension, but did not differ in the temporal dimension. In this case thus all information was contained in the spatial dimension. In case 3 (S10D Fig right) the blocks differed in the temporal dimension, but did not differ in the spatial dimension. In this case all information was carried along the temporal dimension. For each of these cases, we then studied the stimulus decoding performance as a function of the number of trials (total number of training and test set trials) per stimulus and SNR of patterns for the non-permuted single-trial population responses (space-and-time information), for the single-trial population responses permuted along the temporal dimension (space-only information) and for the single-trial population responses permuted along the spatial dimension (time-only information).

We found (S10E Fig) that for case 1 (constructed to contain information in both space and time, S10A Fig), both space-only information (left column, center panel) and time-only information (left column, bottom panel) were below the full space-and-time information (left column, top panel) for all considered SNR values and number of trials. This means that our method correctly found over the whole parameter space the correct

result that there was genuine information both in the spatial and in the temporal dimension that could not be possibly recovered from the other dimension.

For case 2 (constructed to contain information in space only, S10B Fig), space-only information (S10E Fig, center column, center panel) was statistically indistinguishable (p = 0.5, paired t-test over bins) from the space-and-time information (S10E Fig, center column, top panel). In contrast, in this case time-only information (S10E Fig bottom center panel) was down to chance level (16.7% for 6 stimuli), indicating that space-by-time NMF coupled with the permutation procedure correctly ruled that the temporal dimension did not carry information in this case.

Similarly, for case 3 (constructed to contain information in time only, S10C Fig), time-only information (S10E Fig, right column, bottom panel) and space-and-time information (S10E Fig, right column, top panel) were statistically indistinguishable (p = 0.82, paired t-test over bins), whereas space-only information (S10E Fig, right column, center panel) was down to chance level, meaning that space-by-time NMF coupled with the permutation procedure correctly ruled that the spatial dimension did not carry information in this case.

Thus, space-by-time NMF coupled with the permutation procedure correctly detected the respective role of space and time in information coding when tested on these data.

Signal and noise correlations of neurons that take part in the same or different spatial module

We calculated signal and noise correlations in order to quantify the relation between neural dependencies and identified modules. To do so, we counted the number of spikes of each neuron over the whole analyzed trial (300 ms) and calculated Pearson correlation coefficients between these spike counts. Signal correlations were calculated for each pair of neurons over all trials and stimuli and then averaged over all pairs. Noise correlations were calculated separately for each stimulus and each pair and then averaged over all stimuli and pairs.

We compared signal and noise correlations between two groups of neuron pairs: the "within modules" group representing pairs of neurons belonging to at least one common module and the "across modules" group representing all pairs of neurons. We used the following procedure to decide whether a pair of neurons belongs to the "within modules" group:

For a given threshold t:

1. Enumerate all pairs of neurons (i, j) with $i > j$.

2. Pair (i, j) belongs to the "within modules" group if there is a spatial module $\mathbf{b}_k^{spa}$ for which $\mathbf{b}_k^{spa}(i) > t$ and $\mathbf{b}_k^{spa}(j) > t$.

There was no objective and unique threshold to decide whether a pair should be in the "within modules" group. The selection of the threshold therefore was an arbitrary choice. We decided to optimize the threshold t such that the number of pairs in the "within modules" group was exactly half the total number of pairs. In order to obtain a conservative estimate of the impact of module affiliation, we then randomly drew half the total number of pairs from the pool of all neuron pairs to form the "across modules" group and calculated signal and noise correlations separately for both groups.

We found (S15 Fig) that both noise (S15B Fig) and signal (S15C Fig) correlations were positive and significantly larger for within-modules pairs of neurons than for across-

modules pairs of neurons (one-tailed t-test, $p < 0.001$). The stronger signal and noise correlations in the “within modules” group indicate that spatial NMF modules represent ensembles of neurons that tend to fire together. This intuition is visually supported by S15A Fig, which shows raster plots of RGC activity within and across modules in response to a small set of stimuli. Neurons within modules tend to have more similar firing patterns and more consistent stimulus specific responses than neurons that do not share a common spatial module.

Investigating the ceiling effect in decoding performance with the natural image datasets

For the experimental retinal dataset obtained in response to natural images, we found that using space-only information led to a very small but significant decrease in decoding performance compared to using space-and-time information. This may partly be explained by a ceiling effect: for the image datasets, the task may just be too simple to extract any difference in performance with and without temporal information. Decoding performance is already at almost 100% when using space-only information. Therefore, using space-and-time information could not lead to much greater decoding accuracy, resulting in a small performance drop for the time-only code. If the decoding accuracy using space-and-time information was lower, however, the ceiling effect should vanish and the space-only loss should increase.

We investigated this idea in S12 Fig where we show that performance loss for shuffling in time was greater when decoding performance did not saturate on the image datasets. To bring the decoding performance into a non-saturating regime, we divided the population in each session into a fraction ($1/8^{th}$, $1/4^{th}$, and 1/2) of the total population size and evaluated decoding accuracy using only the subpopulation in the space-by-time NMF and the LDA classification. The procedure for the performance evaluation was the same as before except for the reduced population size. Each comparison between space-only, time-only and space-and-time information was evaluated at the same population size (e.g. we compared space-and-time information of $1/8^{th}$ subpopulation with space-only and time-only information of the same $1/8^{th}$ subpopulation).

We found that when using time-only information, performance loss stayed relatively constant between 77% and 80% irrespective of the population fraction. When using space-only information, on the other hand, the drop in performance increased with decreasing population size and reached 23% loss when we reduced the population to $1/8^{th}$ of its original size. These results are consistent with the idea that the small drop in performance in the space-only condition was due to a ceiling effect.

Alternative initialization procedures

Besides the random initialization procedure detailed in step 1), we also tried random initializations with multiple replicates and variants of Nonnegative Double Singular Value Decomposition (NNDSVD, [2]) on the natural image datasets. We compared convergence speed and reconstruction error after 2000 space-by-time NMF iterations. We found increased convergence speed when using the alternative initialization methods, with the NNDSVD method converging particularly fast, but the final modules and reconstruction error were independent of the initialization method.

Alternative noise models

Tucker-2 rules that minimize the Frobenius norm - like the ones we present here - have an implicit Gaussian noise assumption. This is the simplest and most common assumption for NMF-based and other update rules [3-5]. We also applied a variant of space-by-time NMF that utilizes a Poisson noise assumption. On the retina datasets that we analyzed with the Poisson noise assumption (natural images and natural movies), we found no significant difference between the decoding performance for the Gaussian noise assumption (corresponding to the methods we present in this paper) and the performance for the Poisson noise assumption (Wilcoxon rank sum test, $p = 0.37$).

Firing rate normalization

The objective functions of the Tucker-2 algorithms such as our space-by-time NMF and the orthogonal Tucker-2 method minimize the Frobenius norm and therefore favor reconstruction of neurons with higher firing rates. In all the analyses that we present in the main paper, we did not normalize the firing rate of neurons and thus accepted that neurons with lower firing rates may not be reconstructed as well as those with higher firing rates. Note, however, that this may be desirable if neurons with higher firing rates carry more information (as it was the case in our data, since we verified that there was a positive correlation between a neuron's mean firing rate and its sensory information, result not shown) and if the major components of a "firing pattern" that we want to reconstruct are those from the neurons that fire the most.

To check whether normalization could improve decoding performance, we normalized the overall number of spikes of each neuron over all trials and stimuli. We then applied our space-by-time NMF method to the normalized responses of the natural images and movies datasets. On all of these datasets, we found a decrease in decoding performance when analyzing the normalized responses compared to when analyzing the original responses. On the image datasets, average decoding performance decreased by 5.9%, and on the movie datasets, average decoding performance decreased by 19.1%.

Activation coefficient sparseness

We quantified sparseness of the activation coefficient matrices by applying the sparseness measure introduced in [6]. For the natural image datasets, the natural movie datasets, the flashed gratings dataset and the shifted natural image datasets the average sparseness of the activation coefficient matrices over trials and sessions amounted to 64.2%, 79.4%, 43.3% and 61.3%, respectively.

Selection of optimal number of modules

The correct underlying number of modules is unknown in experimental data and must be determined empirically. In the main text, we propose to select the optimal number by optimizing cross-validated accuracy on the test set. There is one concern with this approach that we will discuss in the following.

Our goal was to compare different methods, i.e. we wanted to know whether space-by-time NMF performs better than spatiotemporal NMF, PCA, ICA and FA. The comparison must not be biased by overfitting. Overfitting occurs and affects the comparison if we have an unreasonably flexible model that can fit not only the signal but also the noise and if we use the same dataset (and therefore the same noise) to evaluate performance. The performance measure will then be biased towards the model that fits the noise of

that particular dataset very well, but that model might perform very badly on another dataset with different noise.

To avoid the overfitting bias, we used a training/test set procedure. The idea was to fit the parameters of the model to the training set and to evaluate performance on a separate test set. In this case, the test set noise could not affect the model parameters. However, the numbers of modules, number of principle components etc. are in fact parameters of the model. Therefore, we must not use the test set for selecting the numbers of modules. Otherwise we would contaminate the model parameters with noise from the test set and could potentially get an overfitting bias: the number of modules might be such that the model fits the noise in the test set particularly well. Again, the method comparison then does not necessarily generalize well to other datasets with different noise.

To completely avoid this problem, we could use a leave-one-out procedure on the training set for selecting the numbers of modules/principle components etc. Here, we would not touch the test set at all for selecting the numbers and instead select the number of modules as well as the modules from the training set only.

Let us call our original training set A_0 and our original test set E_0. In the main text, we propose to use LDA-decoding performance on E_0 as a performance measure: for a given number of modules, we fit the modules to A_0 and evaluated decoding performance on E_0. This can be done for all possible numbers of modules and then the number with the best performance on E_0 can be selected.

In the leave-one-out procedure, we set aside E_0 and did not use it for selecting the modules at all. Instead we took out one trial from the training set A_0. This single trial is our new test set E_1. The remaining trials formed our new training set A_1. Then we performed exactly the same procedure on A_1 and E_1 that, according to the proposal in the main text we would perform on A_0 and E_0: For a given number of modules, we fit the modules to A_1 and evaluated LDA-decoding performance on E_1 (on a single trial). We saved the performance result in p_1. Then we put the trial from E_1 back into our training set and took out the next trial which yielded different sets A_2 and E_2. Again, we performed the same procedure and obtain p_2. We did this for all S trials and obtained performance values $p_1, \ldots, p_S$. We averaged over these values to get a single performance value for the given number of modules. This whole procedure was performed for each number of modules and we selected the number of modules with the optimal performance.

We then had an optimal number of modules without having looked at E_0. To finally compare the different methods, we returned to our original sets A_0 and E_0. We took the optimal number of modules and fit these modules to A_0. Then we computed LDA-decoding performance on E_0. We applied this whole procedure for each of the methods (space-by-time, PCA, ICA etc.) and compared the decoding performance. This comparison is completely free from any overfitting bias, since the test set was not used for any model fitting.

In S4A Fig, we report decoding performance averaged over all leave-one-out trials of the training set of spatiotemporal NMF obtained when simulating 30 trials per stimulus for different numbers of modules. In these examples, we achieved the best average decoding performance with the ground truth number of modules (marked with a white square). These results demonstrate the effectiveness of the procedure on simulated data.

For the natural data, it turned out that the numbers of modules, principle components etc. that we obtained from A_0 and E_0 were exactly the same as the ones that we obtained from the leave-one-out procedure on A_0 only. Apparently, the characteristics in the data that determine the number of modules were the same in A_0 and E_0. Since the two selection methods yielded the same results and nothing else changed in the analysis, we report the results for the simplified procedure in the main text.

Rank order decoder

For the flashed gratings dataset, we also evaluated the performance of the rank order decoder. This decoder is based on the relative order of the first-spike latency of each neuron [7-10]. We applied the same procedure that was used in [8] which is also very similar to that of [7]: On each trial, we ranked neurons according to their first-spike latency (using the MATLAB's *tiedrank* function). For each grating phase, we then constructed a template of the population rank order. We did this by first calculating the mean rank of each neuron over the training trials and then we calculated the tie-corrected rank order of the means. For each test trial, we also ranked neurons according to their first-spike latency and then calculated the Spearman rank correlations between the test trial rank order and each template. We selected the grating phase with the highest correlation as the decoder output.

Supplemental References

1. Tenenbaum JB, De Silva V, Langford JC. A global geometric framework for nonlinear dimensionality reduction. Science. 2000;290(5500):2319-23.
2. Boutsidis C, Gallopoulos E. SVD based initialization: A head start for nonnegative matrix factorization. Pattern Recogn. 2008;41(4):1350-62.
3. Févotte C, Cemgil AT, editors. Nonnegative matrix factorizations as probabilistic inference in composite models. Proc EUSIPCO; 2009.
4. Lee DD, Seung HS. Learning the parts of objects by non-negative matrix factorization. Nature. 1999;401(6755):788-91. doi: 10.1038/44565.
5. Lee DD, Seung HS, editors. Algorithms for non-negative matrix factorization. Advances in Neural Information Processing Systems; 2001.
6. Hoyer PO. Non-negative matrix factorization with sparseness constraints. J Mach Learn Res. 2004;5:1457-69.
7. Johansson RS, Birznieks I. First spikes in ensembles of human tactile afferents code complex spatial fingertip events. Nat Neurosci. 2004;7(2):170-7.
8. Panzeri S, Diamond ME. Information carried by population spike times in the whisker sensory cortex can be decoded without knowledge of stimulus time. Front Synaptic Neurosci. 2010;2.
9. Saal HP, Vijayakumar S, Johansson RS. Information about complex fingertip parameters in individual human tactile afferent neurons. J Neurosci. 2009;29(25):8022-31.
10. Van Rullen R, Thorpe SJ. Rate coding versus temporal order coding: what the retinal ganglion cells tell the visual cortex. Neural Comput. 2001;13(6):1255-83.
