# Supplementary figures and images for: Using Matrix and Tensor Factorizations for the Single-Trial Analysis of Population Spike Trains
Source: PLoS Comput Biol. 2016 Nov 4;12(11):e1005189. doi: 10.1371/journal.pcbi.1005189 (PMC5096699; doi:10.1371/journal.pcbi.1005189)

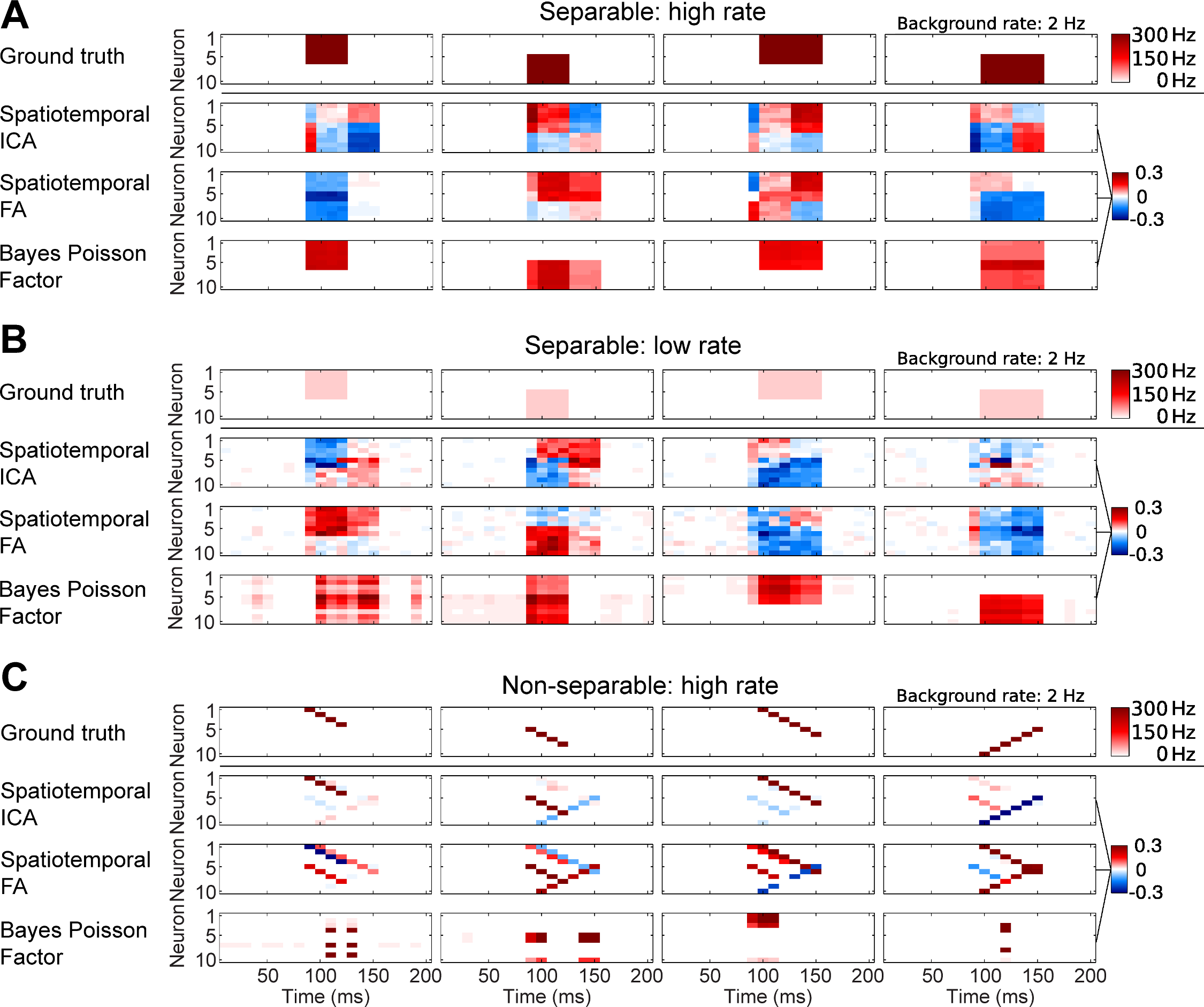

Supplement: S1 Fig — Figure conventions as in Fig 2. (A) A case when the ground truth modules can be factorized into space and time. Top row: Four ground truth modules for generating spike trains. Inhomogeneous Poisson spike trains are generated with a background rate (white) and a stronger foreground rate (red). The red blocks fire with high SNR (300 Hz vs. a background rate of 2 Hz). Each row shows the modules that were recovered by the denoted method. (B) As in panel A but with ground truth patterns made of blocks with lower SNR (30 Hz vs. background rate of 2 Hz). (C) A case of decomposition of high firing rate patterns that are not separable in space and time. (TIF) [file pcbi.1005189.s002.tif]

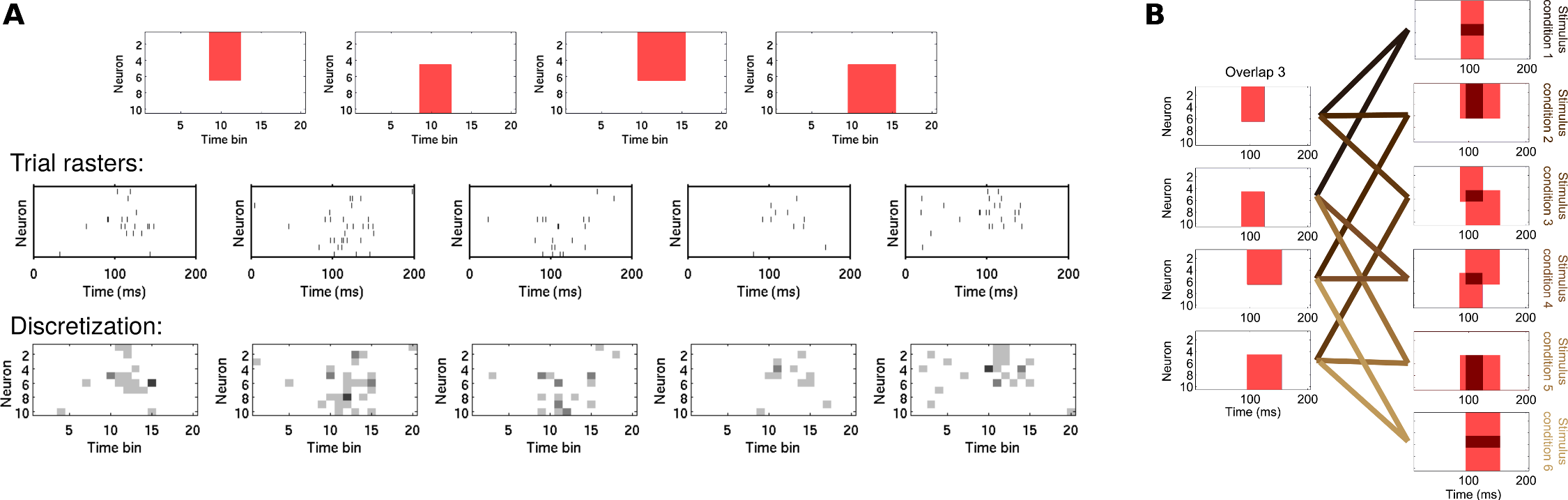

Supplement: S2 Fig — (A) Inhomogeneous Poisson spike trains are generated with a background rate (white) and a stronger foreground rate (red). The foreground blocks appear randomly and (possibly) together. In the shown example, the overlap is 32.25%. Middle panels show example raster plots. Each raster plot represents one trial. Bottom panels show discretization of the corresponding spike raster plots. The number of spikes in each bin is counted to produce the gray value blocks. (B) Patterns of overlapping blocks are combined to create 6 different stimulus conditions. Blocks from overlap 3 (Fig 4A) are shown in the left column. In each stimulus condition (right column), exactly two blocks are present. The selection of blocks is constant for a given stimulus and therefore characterizes the stimulus condition. (TIF) [file pcbi.1005189.s003.tif]

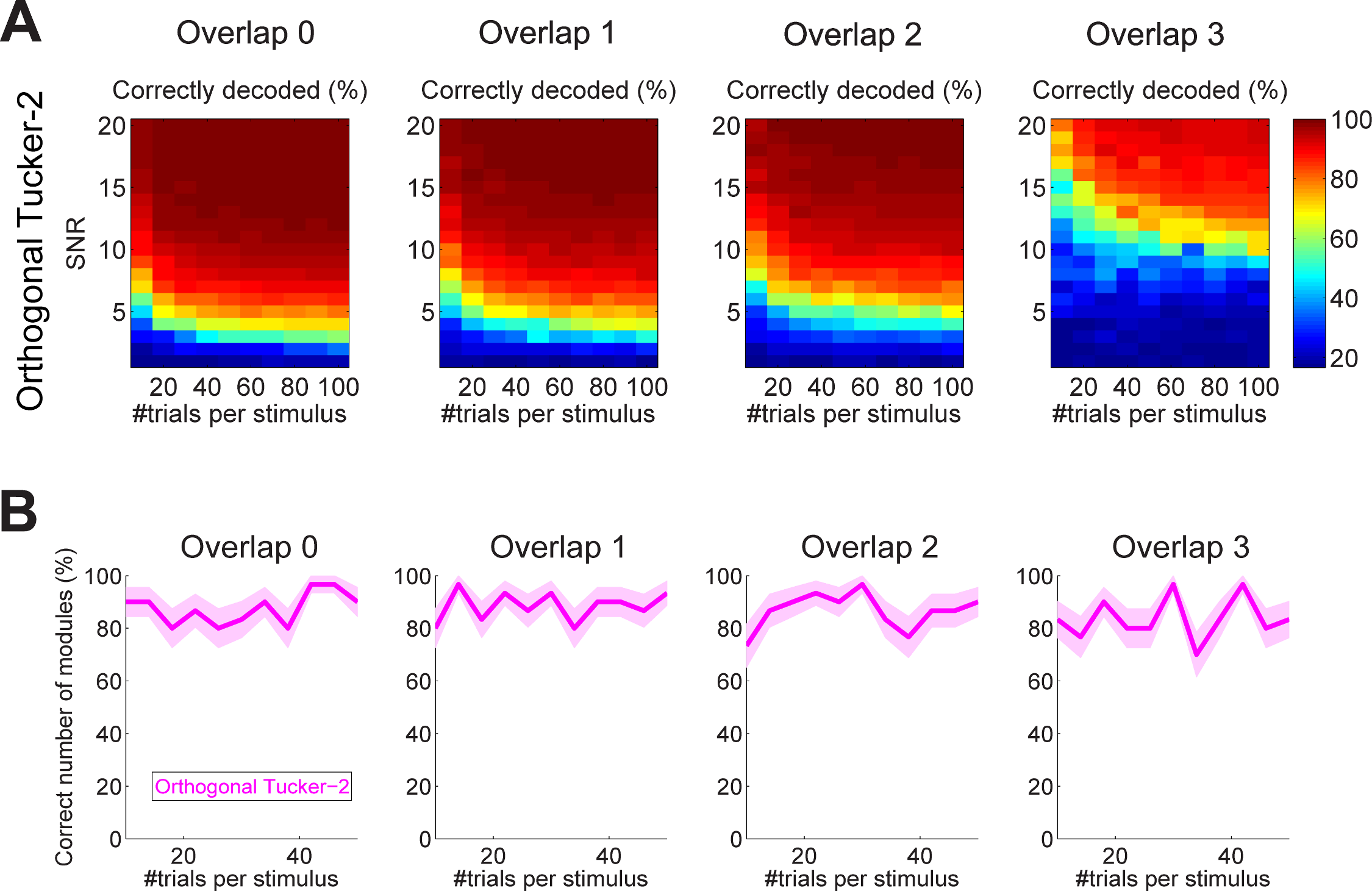

Supplement: S3 Fig — (A) Stimulus decoding performance on simulated data constructed like in Fig 4A and 4B with varying number of trials per stimulus and signal-to-noise ratio (SNR) obtained using orthogonal Tucker-2. (B) Percentage of correct selection of the number of modules as a function of the number of trials per stimulus for orthogonal Tucker-2. We selected the smallest numbers of modules with the maximum test set decoding performance and compared the selected numbers to the ground truth numbers (2 temporal and 2 spatial modules). (TIF) [file pcbi.1005189.s004.tif]

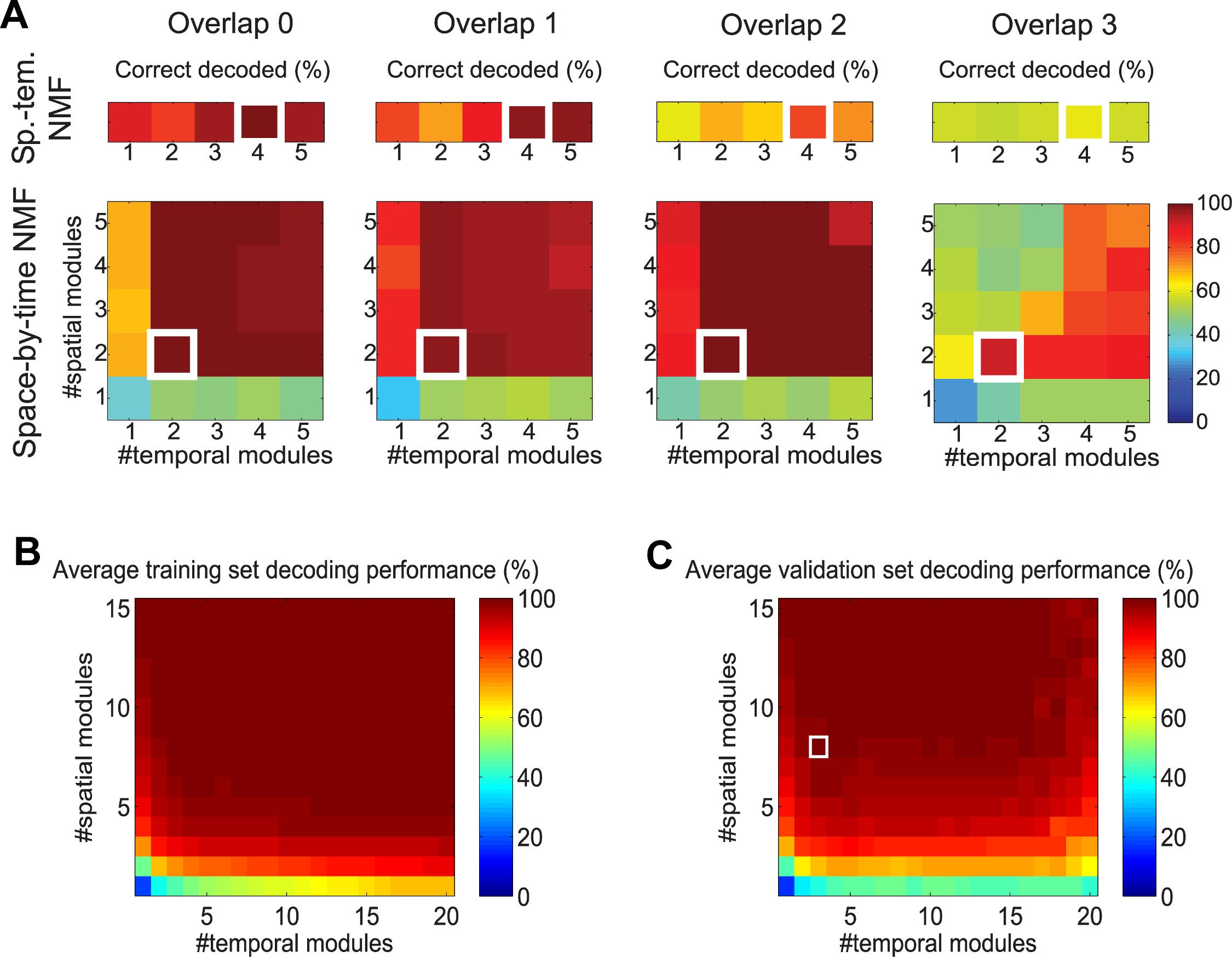

Supplement: S4 Fig — (A) Average leave-one-out validation set decoding performance with SNR = 20 and number of trials (training+test) per stimulus = 30 for spatiotemporal NMF (top row) and for space-by-time NMF (bottom row) is shown. Overlap of the patterns is increasing from left to right (overlap 0 to 3). We select the smallest numbers of modules with the maximum validation decoding performance (white squares—also corresponding to the ground truth: 4 spatiotemporal modules, 2 temporal and 2 spatial modules). (B) Average decoding performance for an example experimental session on the training set averaged over leave-one-out cross-validation sub-samples for different numbers of temporal modules (x-axis) and spatial modules (y-axis). (C) Like B but for the validation set. The smallest numbers of modules with the maximum average validation set performance are selected (white square: 8 spatial modules, 3 temporal modules). (TIF) [file pcbi.1005189.s005.tif]

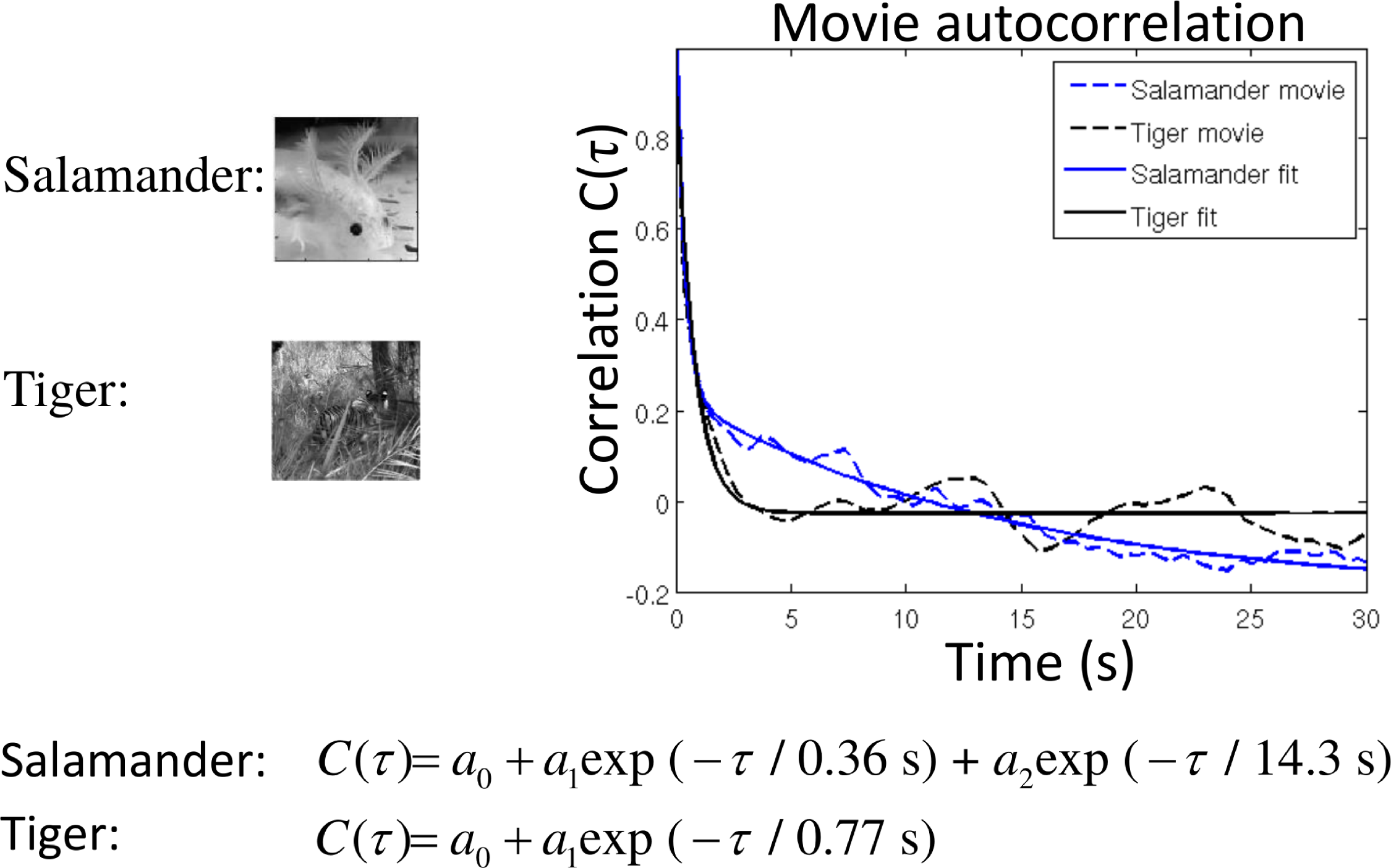

Supplement: S5 Fig — (Top left) Trial still frames from both movies. (Top right) Autocorrelation of the salamander movie (dashed blue) and tiger movie (dashed black) and corresponding salamander and tiger movie autocorrelation fits (solid blue and black, respectively). (Bottom) Equations of the autocorrelation fits. (TIF) [file pcbi.1005189.s006.tif]

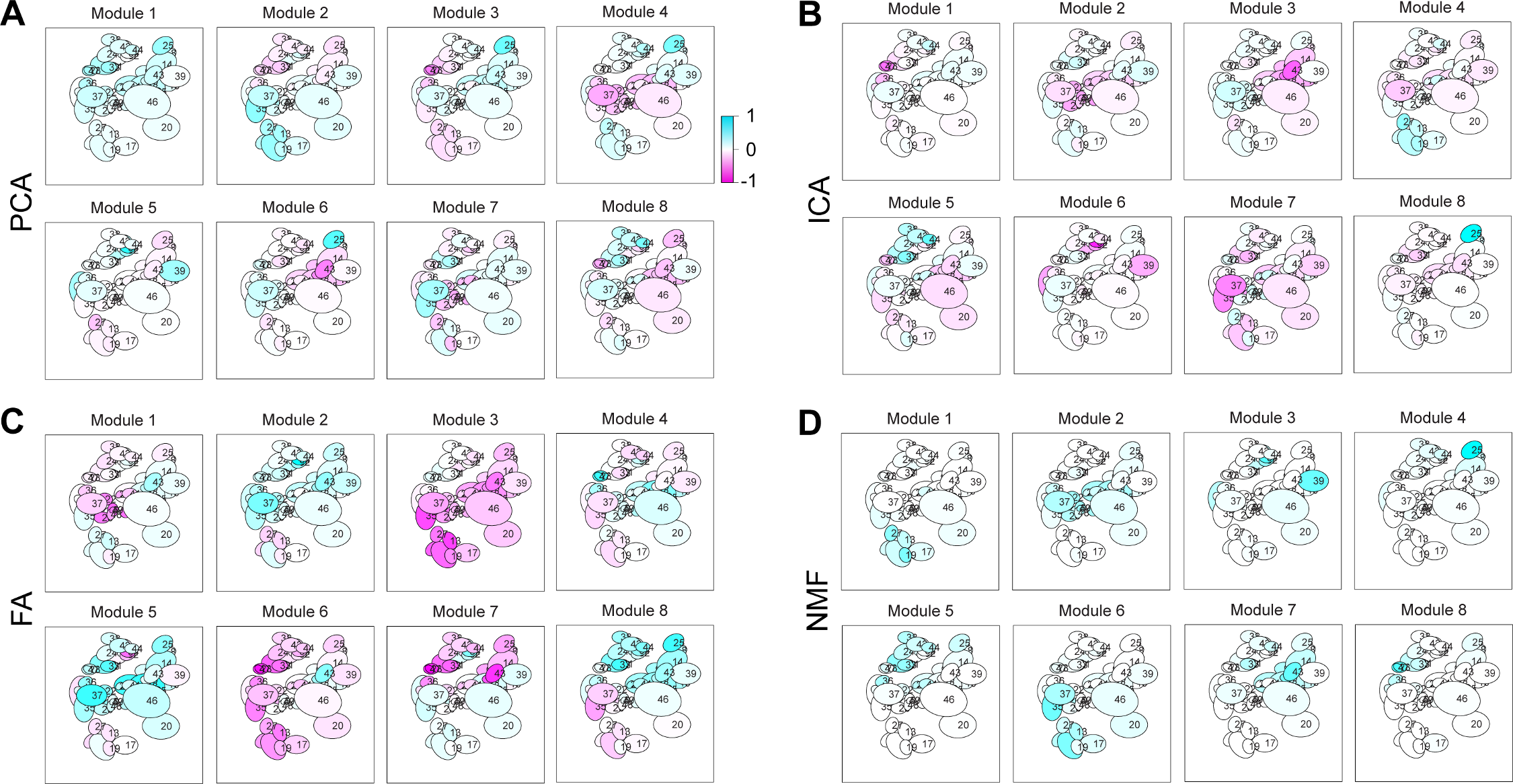

Supplement: S6 Fig — The modules are represented as receptive fields. Cyan represents positive module amplitude and magenta represents negative module amplitude. The more saturated the color the stronger the absolute amplitude of the neuron in the module. (A) Modules identified by PCA. (B) Modules identified by ICA. (C) Modules identified by FA. (D) Modules identified by NMF. (TIF) [file pcbi.1005189.s007.tif]

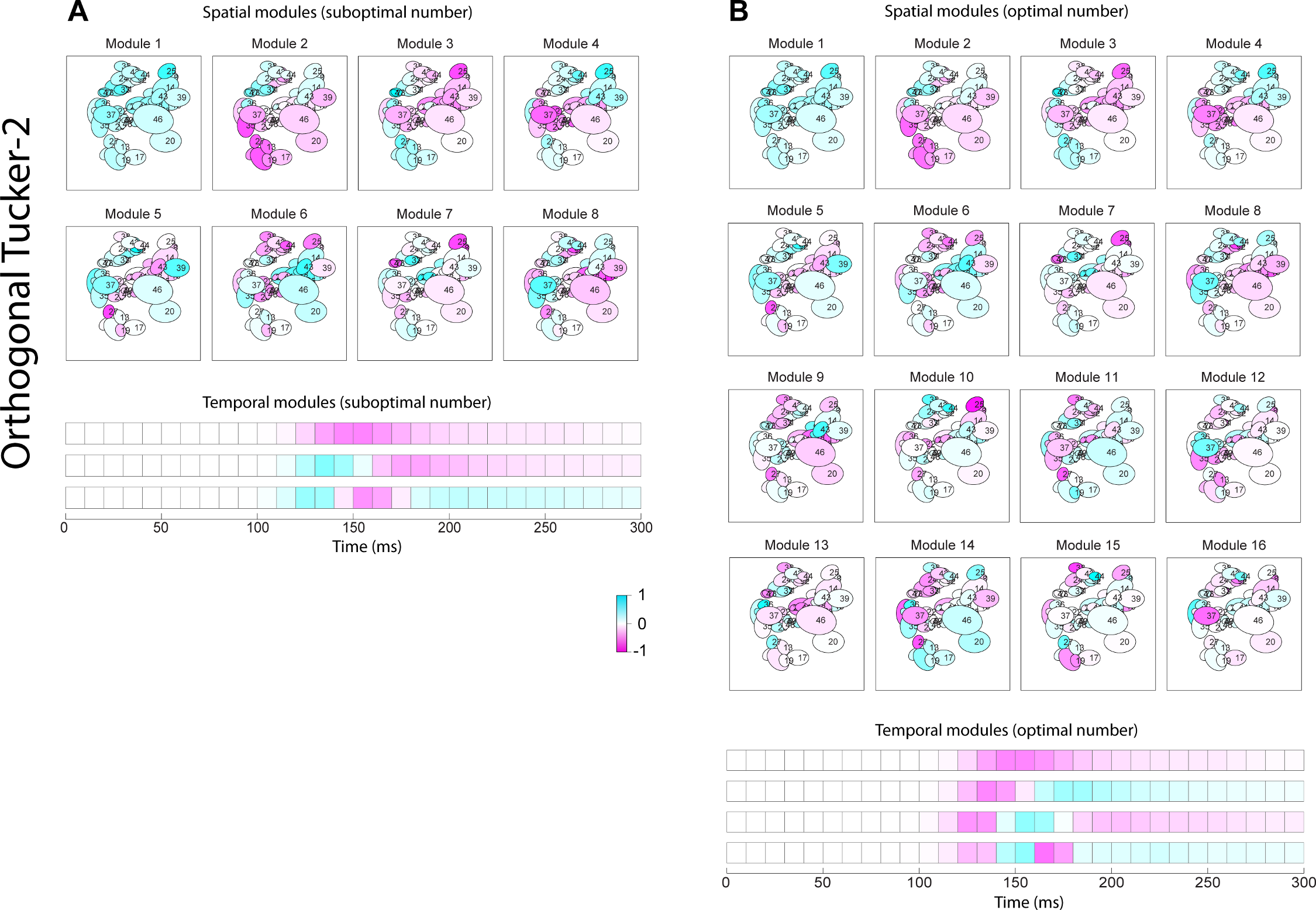

Supplement: S7 Fig — Representation of the modules as in S6 Fig and for the same image dataset. (A) Modules that were identified by orthogonal Tucker-2 for numbers of modules that were optimal for space-by-time NMF to facilitate comparisons with Fig 6 and S6 Fig. (B) Modules that were identified by orthogonal Tucker-2 for numbers of modules that were optimal for orthogonal Tucker-2. The optimal numbers of modules are greater than for space-by-time NMF. (TIF) [file pcbi.1005189.s008.tif]

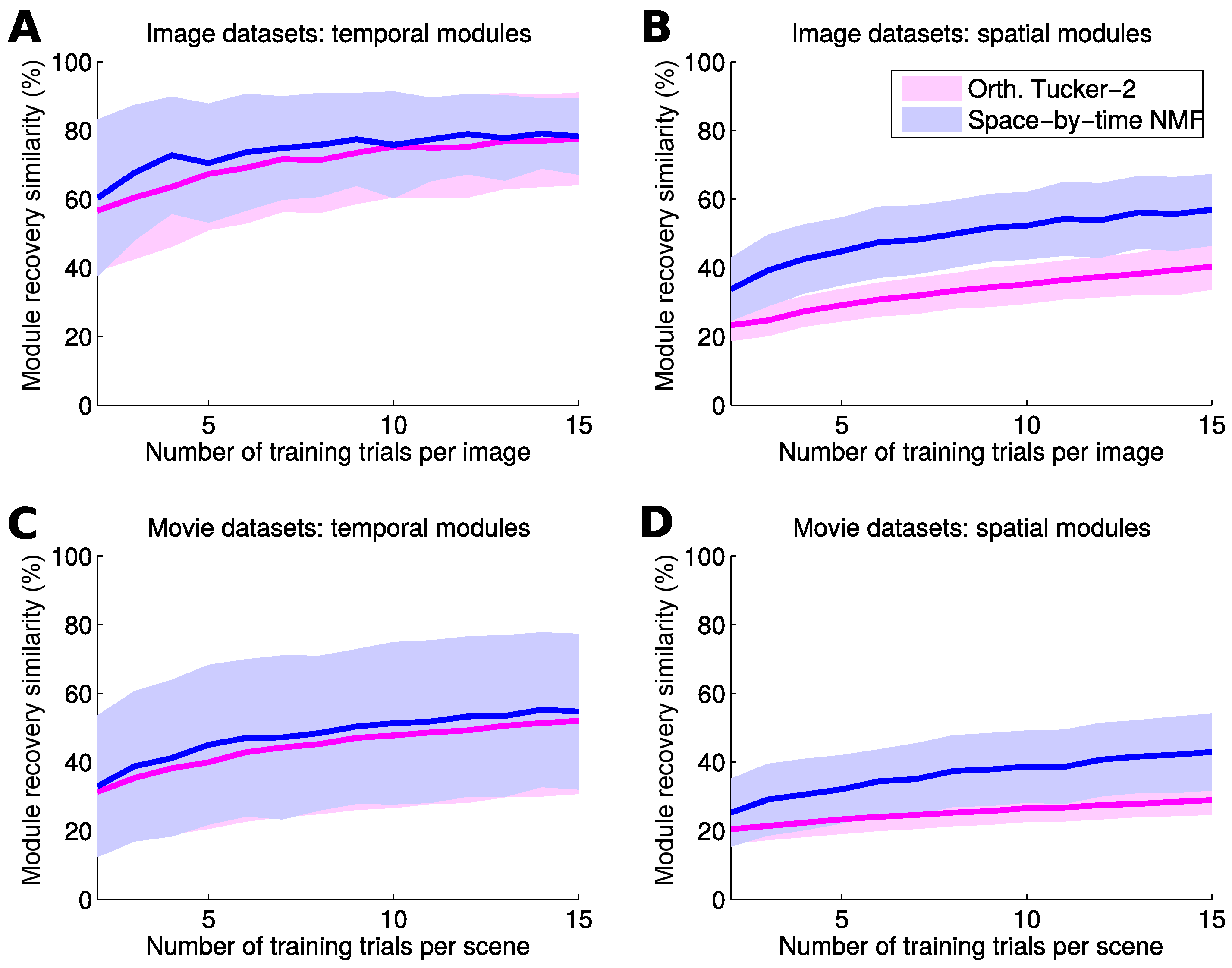

Supplement: S8 Fig — Geodesic similarity between the modules recovered for the full number of trials per stimulus and the modules recovered for a lower number of trials for orthogonal Tucker-2 (magenta) and space-by-time NMF (blue) as a function of the number of trials per stimulus averaged over all image datasets (A, B) or all movie datasets (C, D) for temporal modules (A, C) or spatial modules (B, D). (TIF) [file pcbi.1005189.s009.tif]

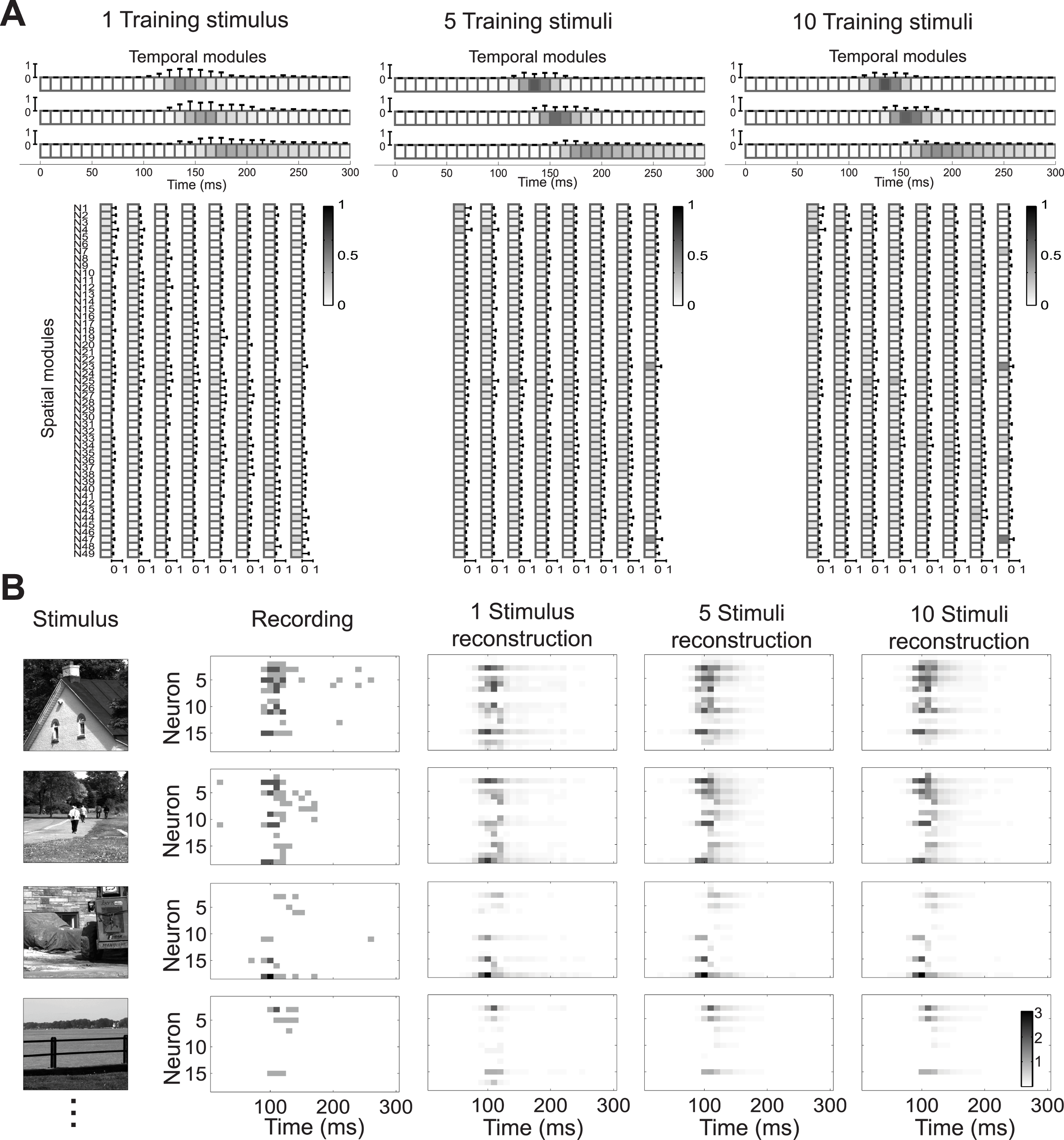

Supplement: S9 Fig — (A) Temporal (top) and spatial (bottom) modules that were obtained by training on data in response to 1 (left), 5 (middle), and 10 (right) training images, drawn randomly from the complete set of 60 stimuli and repeated 10 times to calculate averages and standard deviations. (B) Examples of original trial recordings and reconstructions with different numbers of stimuli for training the space-by-time NMF modules. Neural activity is shown as gray-value bins. The darker the bin the more spikes are present in that bin. Stimuli for the respective row are shown on the left. “Recording” shows the original trials. 1, 5, and 10 stimuli reconstruction show the reconstructions based on space-by-time modules that were trained on 1, 5, and 10 stimuli respectively which do not include the shown stimuli. (TIF) [file pcbi.1005189.s010.tif]

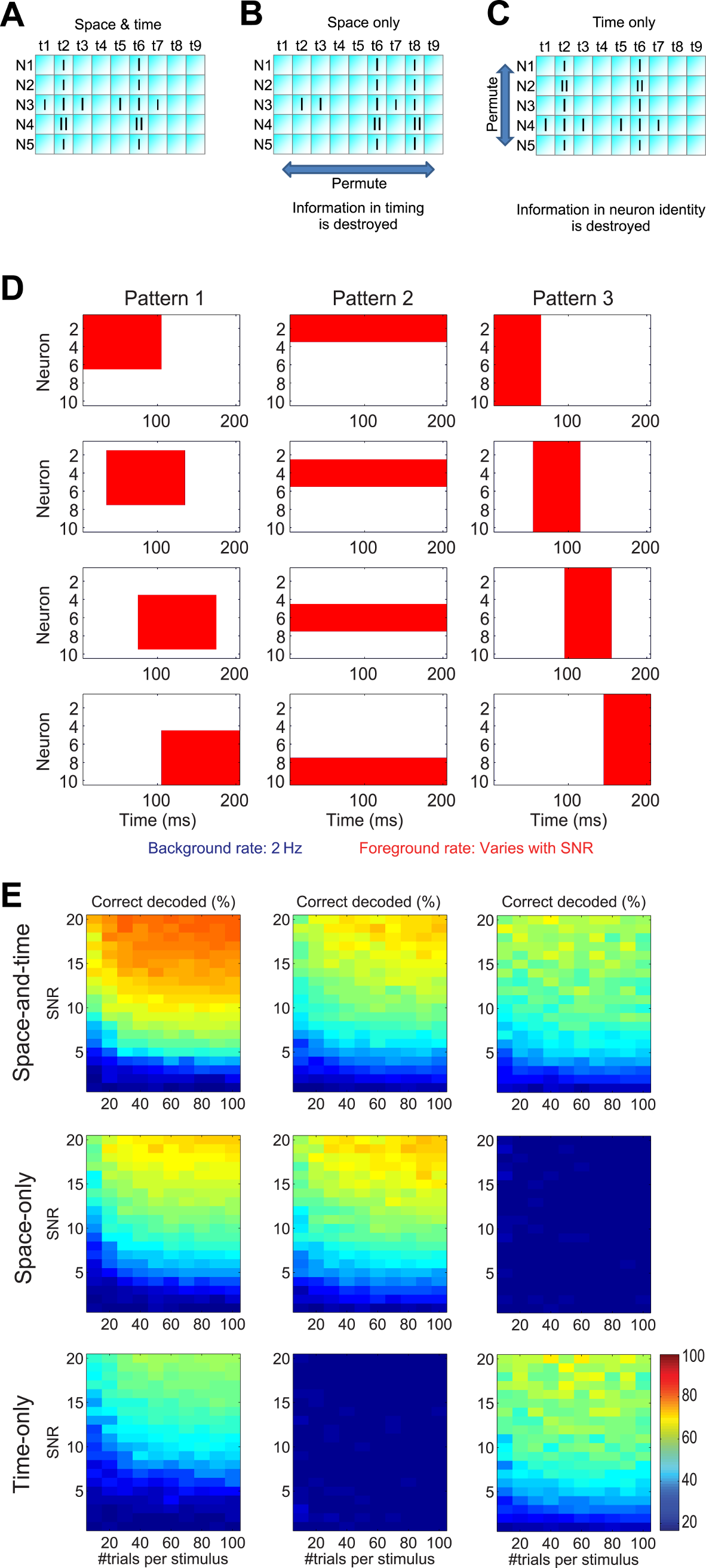

Supplement: S10 Fig — (A) Original spatiotemporal trial. Each block represents a bin. Each line represents a spike. A synthetic population response is shown for 5 neurons N1-5 and 9 time bins t1-9. (B) Space-only condition: responses are permuted across time bins t1-9. Thereby, timing information is destroyed. (C) Time-only condition: responses are permuted across neuron identity. Thereby, spatial information is destroyed. (D) Patterns for spike train generation are plotted with the same conventions as in Fig 4A. Pattern 1 has information in space and time. Pattern 2 has information in space only and pattern 3 has information in time only. The areas of the patterns are kept constant across all three conditions. (E) Stimulus decoding performance of space-by-time NMF on these simulated data with varying number of trials per stimulus and SNR after training on unshuffled responses (space-and-time, top row), after shuffling bins across time (space-only, center row) and after shuffling bins across neurons (time-only, bottom row). SNR as in Fig 4B. (TIF) [file pcbi.1005189.s011.tif]

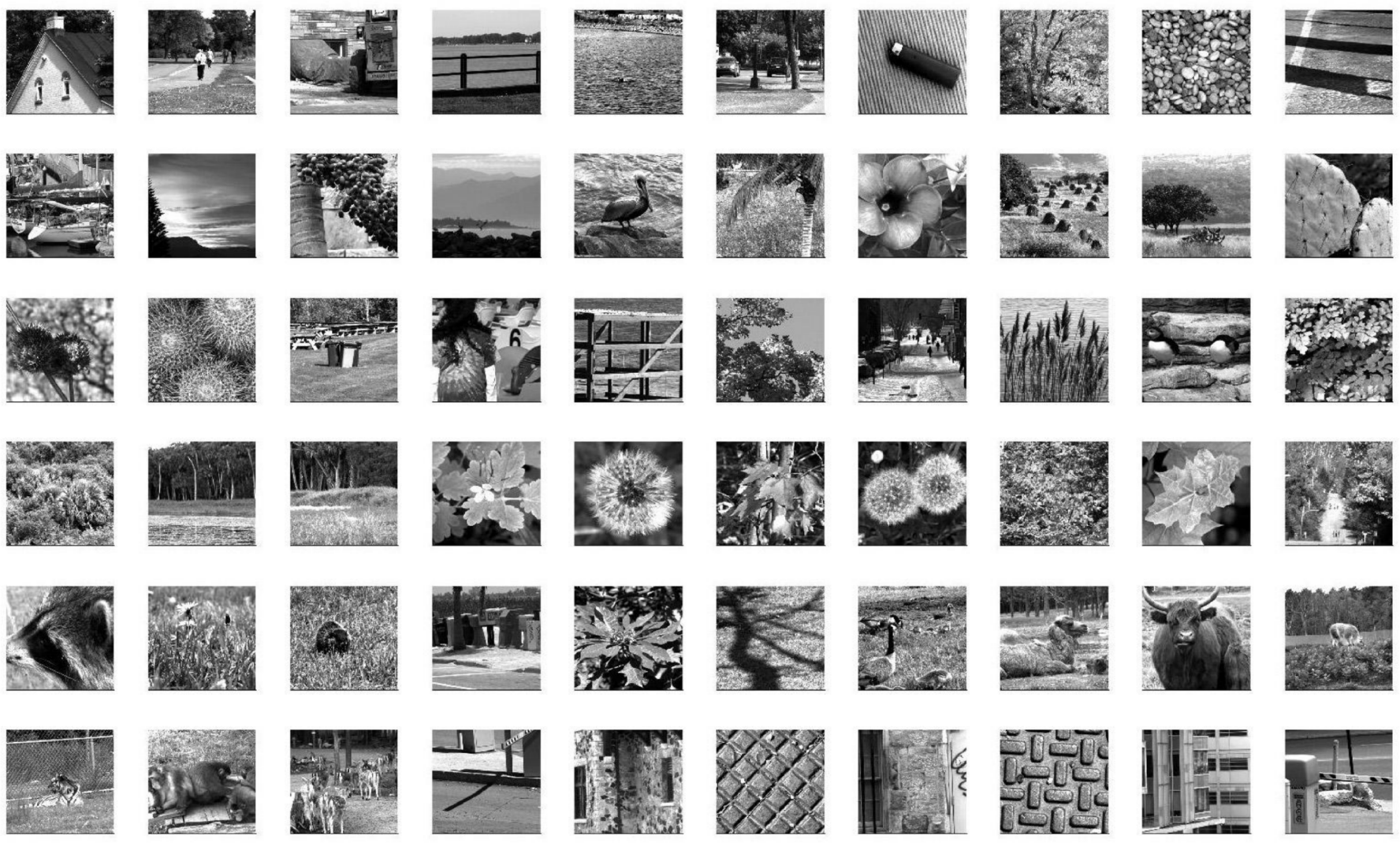

Supplement: S11 Fig — Images were selected from the “McGill Calibrated Colour Image Database” http://tabby.vision.mcgill.ca, converted into grayscale. Mean and standard deviation of the pixel values were normalized for each image. (TIF) [file pcbi.1005189.s012.tif]

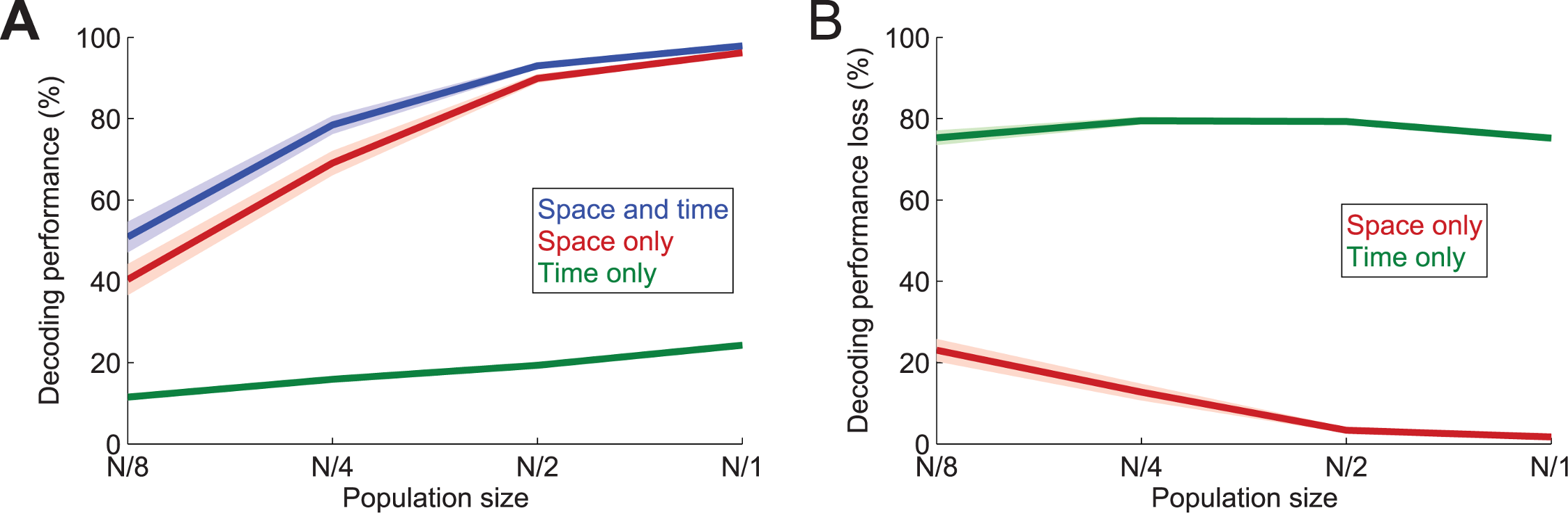

Supplement: S12 Fig — Randomly sampled subsets of the total number of neurons (N) are used for decoding image identities. (A) Decoding performance as a function of the population size for unshuffled responses (space-and-time), responses shuffled across time (space-only) and responses shuffled across cells (time-only). (B) Relative decoding loss as a function of the population size of the respective shuffling method compared to the unshuffled condition. (TIF) [file pcbi.1005189.s013.tif]

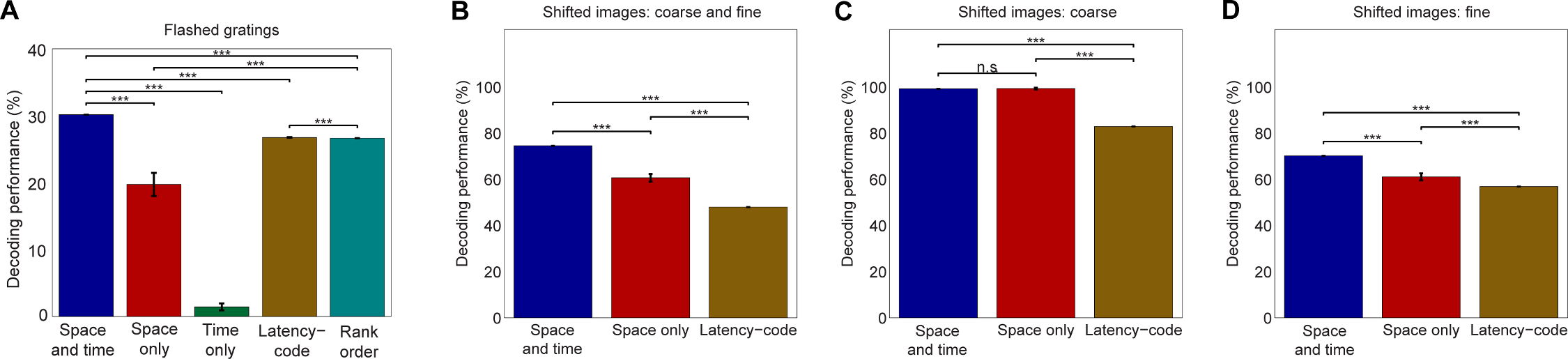

Supplement: S13 Fig — Comparison of decoding performance after training on unshuffled responses with orthogonal Tucker-2 (space-and-time), after shuffling bins across time (space-only), and after keeping only the first spike of each neuron in each trial (latency-code), again after training on responses with orthogonal Tucker-2. We also included the rank order performance from Fig 10D for comparison. (A) Performance on flashed gratings dataset. (B) Performance of decoding image id and image position. (C) Performance of decoding image id for each position, averaged over positions. (D) Performance of decoding image position for each image id, averaged over images. ***p<0.001; two-tailed t-test. Error bars indicate s.e.m. (TIF) [file pcbi.1005189.s014.tif]

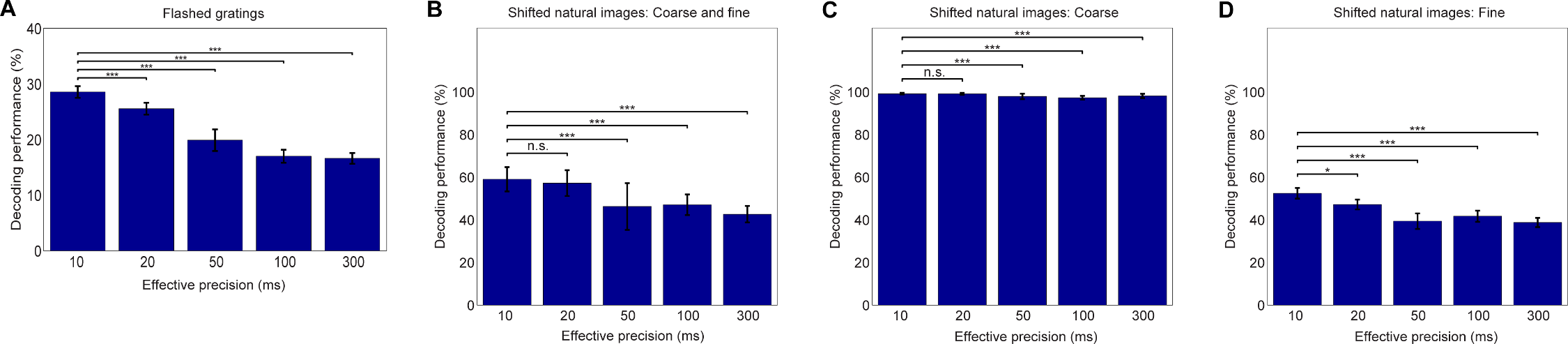

Supplement: S14 Fig — Decoding performance obtained from responses sampled at different effective precisions for the flashed gratings dataset (A) and for the shifted natural image datasets (B-D). The effective precision of 10 ms corresponds to the performance for the unshuffled responses. The effective precisions of 20, 50, 100 and 300 ms were obtained by shuffling bins in 2, 5, 10 and 30 neighboring bins, respectively. (B) Performance of decoding image id and image position. (C) Performance of decoding image id for each position, averaged over positions. (D) Performance of decoding image position for each image id, averaged over images. *p<0.05; ***p<0.001; two-tailed t-test. Error bars indicate s.e.m. (TIF) [file pcbi.1005189.s015.tif]

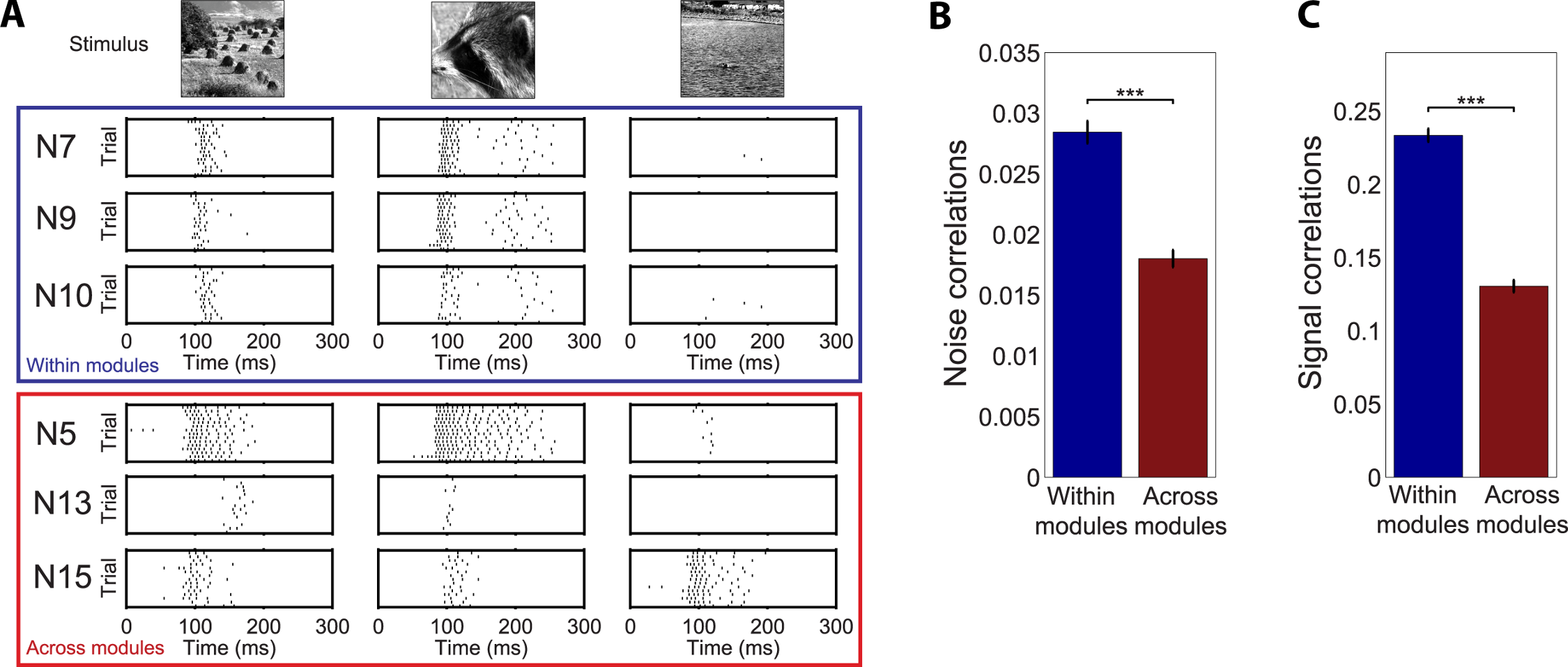

Supplement: S15 Fig — (A) Top: Presented stimuli of the respective column. (Middle) The blue frame marks neurons that belong to the same module (neurons 7, 9, 10). Each row shows raster plots of a single neuron. (Bottom) Raster plots of three neurons belonging to different modules (neurons 5, 13, 15). (B) Noise correlations within and across modules averaged over all image and movie datasets. A pair of neurons is in the “within modules” group if their amplitudes within a module are above a threshold that is set to have half of the pairs in the “within modules” group. ***p<0.001; one-tailed t-test. Error bars indicate s.e.m. (C) As in B, but for signal correlations. (TIF) [file pcbi.1005189.s016.tif]

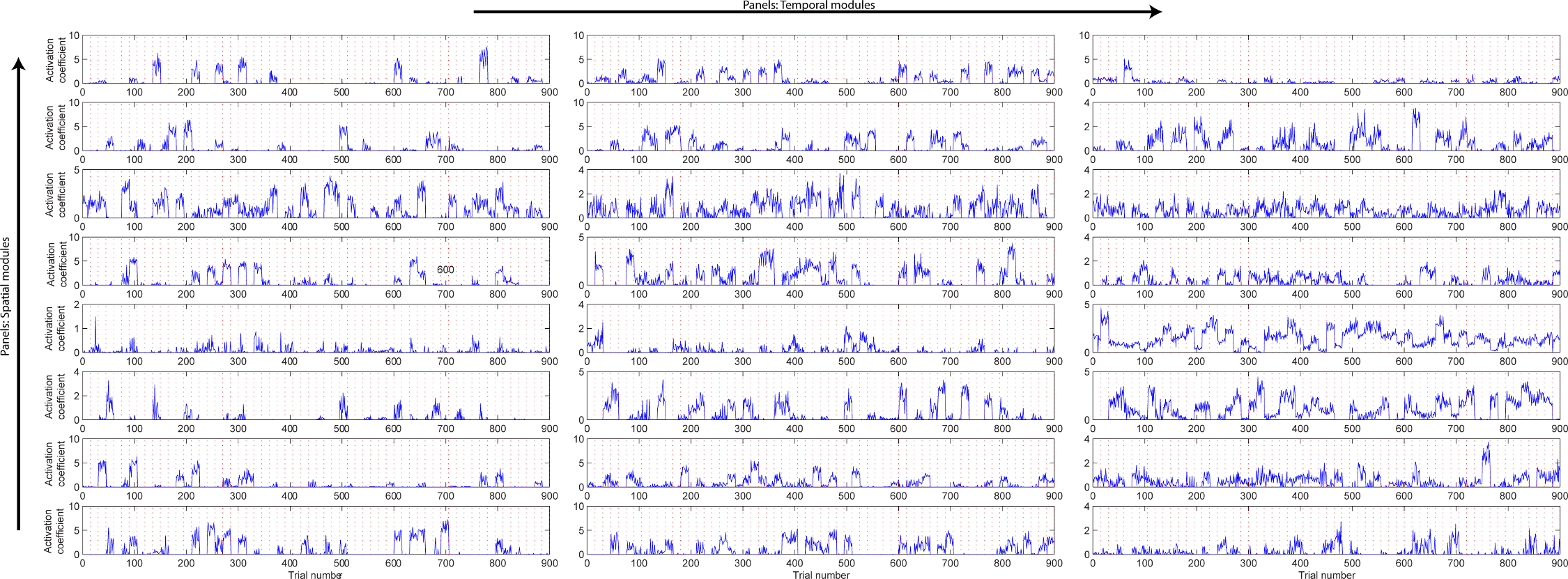

Supplement: S16 Fig — Each panel shows the activation coefficients corresponding to one temporal-spatial module pair (i.e. one element in the Hs matrix) as a function of the trial index s. The dashed red lines separate natural image stimuli: activation coefficients between two dashed red lines belong to trials of the same natural image (15 trials per image in the training set). One can easily appreciate the selectivity of activation coefficients to particular natural images. Many coefficients are zero for particular natural images, indicating moderate sparseness of the coefficient matrices. (TIF) [file pcbi.1005189.s017.tif]

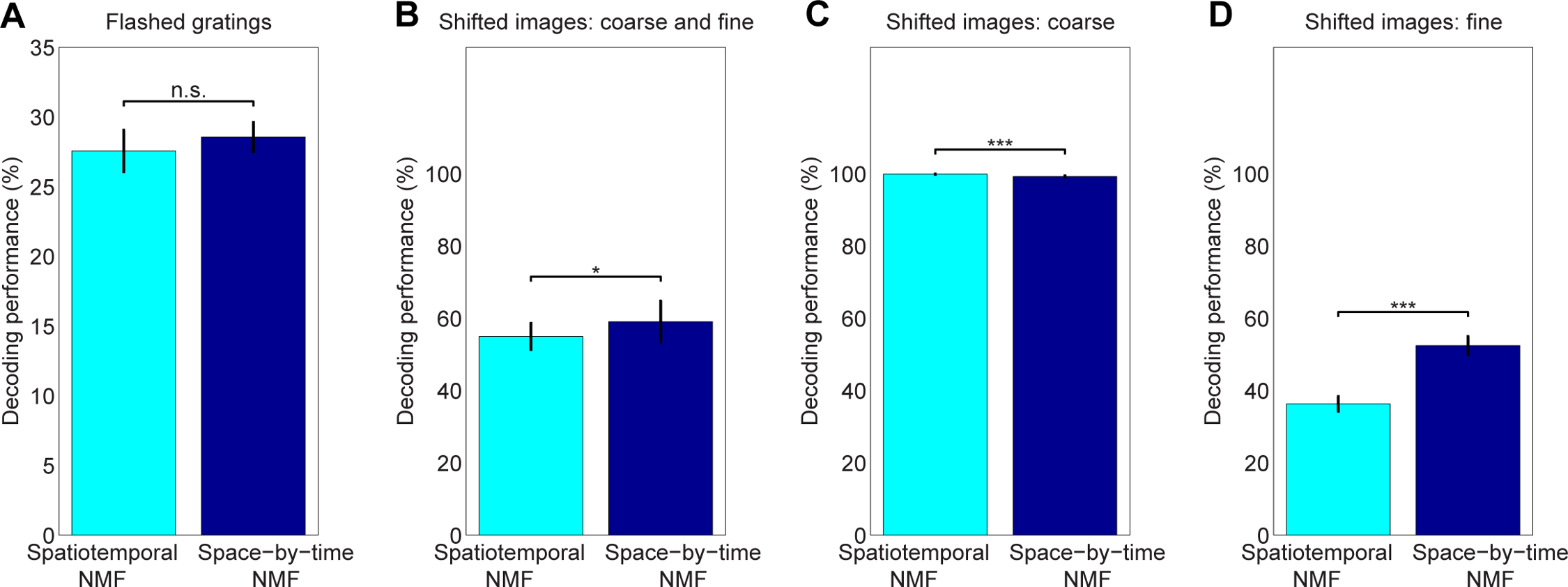

Supplement: S17 Fig — (A) Performance on flashed gratings dataset. (B) Performance of decoding image id and image position. (C) Performance of decoding image id for each position, averaged over positions. (D) Performance of decoding image position for each image id, averaged over images. ***p<0.001; two-tailed t-test. Error bars indicate s.e.m. (TIF) [file pcbi.1005189.s018.tif]
